# Association between comprehensive exposure to multiple occupational hazardous factors and telomere length with hypertension in male steel workers: a case-control study

**DOI:** 10.3389/fpubh.2026.1757027

**Published:** 2026-01-30

**Authors:** Xinyang Chen, Xue Ma, Meng Zhang, Mingyue Liu, Xianghui Xu, Zhenghao Luo, Nan Wang, Jianhui Wu, Ling Xue, Xiaoming Li

**Affiliations:** 1 School of Public Health, North China University of Science and Technology, Tangshan, Hebei, China; 2 Hebei Key Laboratory of Coal Health and Safety, North China University of Science and Technology, Tangshan, Hebei, China; 3 Department of Internal Medicine, North China University of Science and Technology Hospital, Tangshan, Hebei, China

**Keywords:** hypertension, male steel workers, mediation analysis, occupational hazardous factors score, relative telomere length

## Abstract

**Background:**

Steel workers are often exposed to various occupational hazards over the long term, which may be associated with hypertension. Previous studies mainly focused on the relationship between single occupational hazard and hypertension, but the comprehensive effects of multiple occupational hazards and the potential regulation of telomere length are still unclear. This study aims to investigate the relationship between combined exposure to multiple occupational hazards and hypertension in male steel workers, and to assess whether relative telomere length (RTL) plays a mediating role in this relationship.

**Methods:**

A 1:1 matched case-control study was conducted, with cases and controls matched on similar age (±2 years). The study included 350 hypertensive male steel workers and 350 normotensive controls. Occupational hazards [including heat, noise, dust, carbon monoxide (CO), shift work, and occupational stress] and relative telomere length (RTL) were assessed. An occupational hazardous factors score (OHFS) was constructed using the XGBoost model and SHapley Additive exPlanations (SHAP). Conditional logistic regression and quantile regression were used to analyze the associations. Mediation analysis was performed to evaluate the potential mediating effect of RTL in the relationship between OHFS and hypertension.

**Results:**

The risk of hypertension among male steel workers in the higher OHFS groups (24.74~, 38.98~, and ≥56.58) was 1.81, 2.17, and 3.46 times higher than that in the lower OHFS group (< 24.74), respectively (24.74~: *OR* = 1.81, 95% *CI*: 1.14–2.86; 38.98~: *OR* = 2.17, 95% *CI*: 1.39–3.39; ≥56.58: *OR* = 3.46, 95% *CI*: 2.18–5.49). The risk of hypertension among male steel workers in the shorter RTL group was 1.45 times higher than that in the longer RTL group (*OR* = 1.45, 95% *CI*: 1.04–2.03). A significant multiplicative interaction was observed between OHFS and RTL on hypertension (*P* < 0.001). Mediation analysis showed a partial mediating effect of RTL on the association between OHFS and hypertension (proportion mediated: 16.67%).

**Conclusion:**

Among male steel workers, higher OHFS is associated with an increased risk of hypertension, and RTL plays a partial mediating role in the relationship between OHFS and hypertension.

## 1 Introduction

Hypertension, as a common chronic disease, has become the most prevalent cardiovascular disease globally. It is the primary risk factor for severe complications such as heart disease, stroke, and chronic kidney disease, and is also a major risk factor for premature death and disability worldwide (1–3). In the past decade, hypertension has led to approximately 2 million deaths globally (4). With the aging population, changes in lifestyle, and rising obesity rates, the prevalence of hypertension continues to increase. In the China Cardiovascular Health Survey conducted from 2021 to 2022, the weighted prevalence of hypertension among Chinese adults was 31.6% (5). This data highlights the severe threat that hypertension poses to public health and reveals the significant demand for medical resources and disease management.

Hypertension is influenced by both environmental and genetic factors. Previous studies have shown that the prevalence of hypertension among steel workers is 33.89% (6), higher than that in the general population. In addition to behavioral and lifestyle factors, occupational hazards associated with the unique working environment of steel enterprise workers, such as heat, noise, dust, and CO, significantly increase their risk of hypertension (7), making this group a key target for hypertension prevention and control. In previous research, the focus has primarily been on individual occupational hazards (8–10), whereas in real working environments, steel workers are often simultaneously exposed to multiple occupational hazards.

In addition to environmental factors, the occurrence and development of hypertension are also influenced by genetic factors. Telomeres are DNA structures at the ends of chromosomes that protect them from damage and instability. As a biomarker of aging, telomere length has been shown to affect many chronic diseases, including hypertension (11). A prospective study revealed that the average telomere length in hypertensive patients is significantly different from that in individuals with normal blood pressure. After a 5-year follow-up, it was found that individuals with normal blood pressure but short telomeres are more likely to develop hypertension (12). Shortened telomere length may affect the regenerative capacity of endothelial progenitor and myocardial cells, accelerate the aging of vascular endothelial cells, and thereby lead to vascular remodeling and reduced cardiac function, increasing the risk of vascular damage and promoting the occurrence and development of hypertension. Moreover, shortened telomere length may induce a low-grade chronic inflammatory response, elevate pro-inflammatory factor levels, and promote atherosclerosis, thereby affecting blood pressure in the body (13, 14).

Telomere length is susceptible to oxidative stress, and steel workers exposed to various occupational hazards may experience accelerated oxidative damage, which in turn induces changes in telomere length (15). Studies have shown a close relationship between polycyclic aromatic hydrocarbon exposure in coke plant workers and shortened telomere length (16). In addition, research has also found a strong association between environmental lead and cadmium exposure and telomere length (17). Ko et al. (18) also found that the average telomere length of welders was slightly shorter than that of administrative staff. In the future, telomere length may serve as a potential biomarker for predicting cardiovascular diseases in workers exposed to heavy metals.

In summary, the complex occupational hazard environment to which steel workers are chronically exposed may increase the risk of hypertension through mechanisms such as telomere attrition. However, there is currently a lack of systematic studies assessing the comprehensive effects of multiple occupational hazards on hypertension. There is also a lack of research exploring the potential mediating and interactive mechanisms at the molecular level, such as telomere length. Therefore, this study hypothesizes that male steel workers comprehensive exposure to multiple occupational hazards can increase the risk of hypertension, and that telomere length may mediate the association between comprehensive occupational hazard exposure and hypertension. Based on the above hypothesis, this study will conduct a case-control study to measure the telomere length of male steel workers and explore the relationship between comprehensive occupational hazard exposure and hypertension.

## 2 Materials and methods

### 2.1 Study design and population

This study is a case-control study, based on the occupational cohort study of health effects in the Beijing–Tianjin–Hebei region. The study subjects were 1,101 employees of a steel enterprise who underwent occupational health examinations at Hongci Medical Group from July to August 2024. According to the “Chinese Guidelines for the Prevention and Treatment of Hypertension” (2024 revised edition), a total of 485 steel workers were diagnosed with hypertension. After excluding those with a history of liver or kidney disease, those with missing basic information, those who did not undergo biochemical or routine blood tests, and those who had changed job types, 363 steel workers were included in the case group. This study employed a 1:1 matched case-control design. The criteria were as follows: individuals with normal blood pressure were included based on the same hypertension diagnostic criteria used for the case group; the inclusion and exclusion criteria were consistent with those of the case group; and each case was matched to a control by age (±2 years). Because there are only male steel workers in the case group, only male steel workers are included as the research object. Ultimately, 350 case-control pairs were matched. The selection process of the study subjects is shown in Supplementary Figure S1. This study followed the principles of the Declaration of Helsinki and was approved by the Ethics Committee of North China University of Science and Technology (approval number: 16040), and all participants have informed consent and signed the informed consent form. In this study, the efficacy of 1:1 matched case-control study was estimated, and the calculation formula is as follows:


$$\begin{array}{l} Z_{\beta }=\sqrt{\frac{\text{n}\left(P_{1}-P_{0}\right)}{2\bar{\text{pq}}}}-Z_{\alpha }\\ P_{1}=\frac{P_{0}OR}{1-P_{0}\left(OR-1\right)}\\ \bar{p}=\frac{P_{0}+P_{1}}{2}\\ \bar{q}=1-\bar{p} \end{array}$$

The *Z*_β_ values were calculated, and the β values were determined by referring to the standard normal distribution table, with power = 1 – β. The study included a total of *n* = 350 pairs. *P*_0_ represents the proportion of exposure to risk factors in the control group. In this study, the exposure rates for the Q2, Q3, and Q4 groups of occupational hazard factor scores were 23.7%, 25.7%, and 18.5%, respectively, with corresponding odds ratios (*OR*) of 1.81, 2.17, and 3.46. With α = 0.05, the calculated *Z*_β_ values were 8.18, 9.59, and 8.97, respectively. The exposure rate of short telomere length in the control group was 43.7%, OR was 1.45, and the calculated *Z*_β_ value was 6.11. All β values were < 0.001, 1 – β > 0.999. In hypothesis testing, the required statistical power is typically 80% or 90%. The sample size used in this study achieved a power exceeding 90%, thereby meeting the sample size requirements.

### 2.2 Data collection

This study collected data through on-site hygiene surveys, questionnaires, physical examination information, and laboratory test results.

On-site hygiene survey: understanding the production process of the steel industry, systematically investigating the working environment and working hours arrangements of different factories and job types, collecting exposure information on occupational hazard factors such as heat, noise, dust, CO, and shift work.

Questionnaire information: general demographic characteristics (age, gender, marital status, education level, family income), personal and family medical history (hypertension, diabetes), behavioral and lifestyle factors (smoking, drinking, diet, physical activity), work-related information (occupational stress, shift work, exposure to occupational hazardous factors such as heat, noise, dust, CO).

Physical examination: including height, weight, and blood pressure.

Laboratory tests: after fasting for 12 h overnight, venous blood was drawn from the study subjects. Fasting blood glucose, total cholesterol, triglycerides, high-density lipoprotein cholesterol, low-density lipoprotein cholesterol, and other biochemical blood indicators were tested using the Mindray fully automatic biochemical analyzer (BS-800). The tests were conducted in the central laboratory of Hongci Hospital's Department of Laboratory Medicine.

### 2.3 Assessment of occupational hazardous factors exposure

1) Heat (19): according to the national standard “Occupational Exposure Limits for Hazardous Factors in the Workplace Part 2: physical Factors,” temperature should be measured during the hottest season of the year. Considering the specific conditions of steel production facilities, temperature measurements were taken at different workplaces. Three to six measurement points were selected at each workplace, and each point was tested three times. The average value was taken as the final result. Workplaces with an average Wet Bulb Globe Temperature (WBGT) of ≥25 °C are defined as high-temperature working conditions, and workers in such environments are considered to be exposed to high temperature.
2) Noise (20): according to the national standard “Grading of Hazardous Workplaces for Occupational Diseases Part 4: noise,” noise exposure is defined as work where the equivalent continuous sound level reaches or exceeds 80 decibels (A) for 8 h per day or 40 h per week. Workers in such environments are considered to be exposed to noise.
3) Dust (21): in accordance with the national standard “Determination of Dust in the Air of Workplaces Part 1: concentration of Total Dust,” as defined by the Chinese standard (Ministry of Health, January 2007), a qualified testing company was selected to conduct on-site total dust concentration measurements. Workers were considered to be exposed to dust if they were in contact with it. The dust measured in this study represents total dust. The actual daily detection results from the steel company were compared with the data provided by the company to ensure the authenticity and reliability of the information.
4) CO (22): a professional testing company with the necessary qualifications was commissioned to conduct on-site CO detection in accordance with the national standard “Occupational Exposure Limits for Hazardous Factors in the Workplace Part 1: chemical Hazardous Factors.” Workers were considered to be exposed to CO if they were working in an environment where CO was present.
5) Occupational stress (OS) (23): the Chinese version of the Job Content Questionnaire (JCQ) was used to measure job strain. The Chinese version of the JCQ contains 22 items, covering three dimensions: job demands, job control, and social support. The items were scored using the Likert scale, with each item ranging from 1 to 4 points. The total score for each dimension was calculated by summing the scores of the items within that dimension. Higher scores in job demands and lower scores in job control indicate a greater imbalance between job demands and job control, which is associated with higher levels of job strain. Social support acts as a buffer against job strain, with higher scores indicating better social support. The job demand-control ratio (D/C ratio) was calculated using formula . If the D/C ratio of a subject was >1.00, it indicated the presence of occupational stress. If the D/C ratio was less than or equal to 1.00, it indicated the absence of occupational stress.

$$\begin{array}{l} D/C\;ratio=Job\;Demand\;Score/\left(Decision\;Latitude\;Score\;\times \;C\right) \end{array}$$ (1)

where C is the ratio of the number of job demand items to the number of decision latitude items, which is 5/9.

6) Shift work (24): shift work refers to a working hour system in which different workers or teams complete the usual daily tasks of 8–24 h through the handover of shifts. Shift work is in contrast to the normal day shift, with working hours that are not fixed but follow a certain pattern. According to the definition of the International Labor Organization, work between 0:00 and 5:00 is considered night shift work. Shift work that includes this time period is defined as night shift work. In this study, shift work refers specifically to night shift work, Categorized as: never (never worked night shifts), Former (stopped working night shifts for at least 6 months or longer at the time of the survey), Now (have been working night shifts continuously for at least 6 months or longer at the time of the survey).
7) Occupational hazardous factors score (OHFS): using the built-in functions of the XGBoost model to assess the relative importance of various occupational hazardous factors, and accordingly drawing a feature importance plot. By introducing the SHapley Additive exPlanations (SHAP) method, an importance value is assigned to each variable, providing an interpretable hierarchy that demonstrates the influence of each variable relative to the others, thereby quantifying the importance of various occupational hazardous factors in the model. Subsequently, based on the minimum relative importance value as a unit of comparison, multiple occupational hazardous factors are scored according to their hazard levels to obtain individual OHFS for steel workers' exposure to occupational hazardous factors.

$$\begin{array}{l} OHFS=S1+S2+S3+S4+S5+S6 \end{array}$$ (2)

where S1, S2, S3, S4, S5, S6 are the scoring values corresponding to various occupational hazardous factors.

### 2.4 Determination of hypertension

According to the classification criteria of the “Chinese Guidelines for the Prevention and Treatment of Hypertension” (2024 revised edition) (25), hypertension is diagnosed if the clinic blood pressure is ≥140/90 mmHg on three separate occasions without antihypertensive medication. If a patient has a history of hypertension and is currently using antihypertensive medication, even if the blood pressure is below the diagnostic threshold, hypertension should still be diagnosed.

### 2.5 Measurement of telomere length

After completing the routine blood tests and biochemical analyses, the blood samples were aliquoted into EP tubes and transferred to a −80 °C freezer for storage and subsequent testing. Genomic DNA was extracted from peripheral blood leukocytes using the Wizard Genomic DNA Purification Kit from Promega Corporation, USA. The concentration and purity of the extracted DNA samples were measured using a UV spectrophotometer (NANODROP2000C), ensuring that the purity (A260/280 ratio) was between 1.7 and 2.0, and the concentration was ≥20 ng/μl. RTL was measured by real-time quantitative PCR using the GoTaq qPCR Master Mix reagent from Promega Corporation, USA. The telomere primer design was based on the studies of O'Callaghan (26, 27), with the single-copy gene 36B4 selected as the internal reference. The specific primer sequences are shown in Supplementary Table S1. The primers were synthesized by Bomed Company in Beijing. The PCR reaction system and program settings are shown in Supplementary Tables S2, S3. Data were automatically collected and analyzed using the software accompanying the PCR system, and the Ct values of the samples were directly read. The ratio of telomere to single-copy gene was expressed as the T/S value. Relative telomere length (RTL) (relative T/S) was obtained by comparing the T/S value of the sample with that of the standard. In qPCR, *T*/*S* = [2^Ct(Tel)^/2^Ct(36B4)^]^−1^ = 2^−Δ*Ct*^, ΔCt=Ct (Tel)-Ct (36B4), relative T/S=2^−(Δ*Ct*1−Δ*Ct*2)^=2^−Δ*ΔCt*^, where ΔCt1 is the ΔCt value of each sample, and ΔCt2 is the ΔCt value of the standard.

### 2.6 Covariates

General demographic characteristics (age, marital status, education level, income), behavioral and lifestyle factors (smoking, drinking, physical activity, diet), and personal medical history (obesity, diabetes, dyslipidemia) were selected as covariates. More detailed information is provided in Supplementary materials.

### 2.7 Statistical analysis

Establish an Excel database. Quantitative data that conform to a normal distribution are described using the mean ± standard deviation. The differences between the case group and the control group were compared using the independent samples *t*-test. Quantitative data that do not conform to a normal distribution are described using the median (interquartile range), and group comparisons are conducted using the Wilcoxon rank-sum test. Categorical variables are described using proportions, and group comparisons are conducted using the χ^2^ test. RTL is divided into two groups (long and short) based on the median. Exposure groups for heat, noise, dust, CO, shift work, and occupational stress are divided into low-duration and high-duration exposure based on the median of work tenure. Conditional logistic regression was used to explore the relationship between OHFS and RTL with hypertension. Quantile regression was employed to assess the impact of OHFS on RTL. Additionally, RTL (long, short) was combined with OHFS (high, low), and conditional logistic regression analysis was conducted to examine the joint effects. Interaction terms were added to the logistic regression model to evaluate multiplicative interactions. Additive interactions were assessed using the attributable proportion of interaction (AP), relative excess risk of interaction (RERI), and synergy index (SI). In mediation analysis, a mediator variable (M) is hypothesized to mediate the relationship between the independent variable (X) and the dependent variable (Y). The following three basic steps need to be satisfied among the variables: (1) Determine the total effect of the independent variable on the dependent variable. (2) Determine the effect of the independent variable on the mediator variable. (3) Determine the effect of the mediator variable on the dependent variable (controlling for the independent variable). The mediation effect analysis was conducted using the Bootstrap method. The relationships among the independent variable, mediator variable, and dependent variable were established using the lm() function in R software. The mediation model was constructed using the mediate() function to analyze the mediating role of RTL, as shown in Supplementary Figure S2. Finally, multiple sensitivity analyses were performed.

All statistical analyses were conducted using SPSS 23.0 and R (4.5.1). A *P* < 0.05 was considered statistically significant.

## 3 Results

### 3.1 Distribution of basic characteristics of study participants

As shown in Table 1, a total of 350 cases and 350 controls were included in this study, matched 1:1 by the similar age (±2 years). Since the case group consisted only of males, only male steel workers were included as study subjects. The average age was 46.60 ± 7.17 (case group) and 46.61 ± 7.15 (control group), with no difference in the age between the two groups (*P* = 0.481). In addition, the results showed that marital status (*P* = 0.507), income (*P* = 0.861), physical activity (*P* = 0.404), and diabetes (*P* = 0.203) were not significantly different between the two groups. However, there were statistically significant differences in work years (*P* < 0.001), education level (*P* = 0.022), smoking (*P* = 0.011), drinking (*P* = 0.004), DASH score (*P* = 0.002), obesity (*P* < 0.001), dyslipidemia (*P* = 0.020), and exposure to occupational hazards (heat, noise, CO, dust, shift work, and occupational stress) (*P* < 0.05) between the two groups. The distribution of general characteristics of the total population is shown in Supplementary Figure S3. The median RTL in the case group was significantly lower than that in the control group [1.05 (IQR: 0.80, 1.35) vs. 1.19 (IQR: 0.89, 1.71), *P* < 0.001]. The distribution of RTL in the overall population is shown in Supplementary Figure S4, and the distributions stratified by case and control groups are presented in Supplementary Figure S5.

**Table 1:** Distribution of basic characteristics between the case and the control [*n* (%)].

**Variable**	**Case (*n* = 350)**	**Control (*n* = 350)**	***χ^2^/t/Z***	***P***
Age^a^ (Years)	46.60 ± 7.17	46.61 ± 7.15	−0.705	0.481
**Age group (Years)**				
< 45	147 (42.0)	150 (42.9)	0.053	0.819
≥45	203 (58.0)	200 (57.1)		
Work years^b^ (Years)	27.00 (22.00, 34.00)	25.00 (19.00, 32.00)	−3.482	< 0.001
**Education level**				
Low	73 (20.9)	47 (13.4)	7.594	0.022
Medium	188 (53.7)	195 (55.7)		
High	89 (25.4)	108 (30.9)		
**Marital status**				
Unmarried	12 (3.4)	12(3.4)	1.357	0.507
Married	334 (95.4)	330 (94.3)		
Other	4 (1.2)	8 (2.3)		
**Income (Yuan)**				
< 1,500	114 (32.6)	109 (31.1)	0.300	0.861
1,500~	172 (49.1)	172 (49.1)		
3,000~	64 (18.3)	69 (19.8)		
**Smoking**				
Never	134 (38.3)	172 (49.1)	8.947	0.011
Former	19 (5.4)	12 (3.4)		
Now	197 (56.3)	166 (47.5)		
**Drinking**				
Never	195 (55.7)	237 (67.7)	10.921	0.004
Former	5 (1.4)	5 (1.4)		
Now	150 (42.9)	108 (30.9)		
**DASH score**				
< 24	141 (40.3)	99 (28.3)	10.006	0.002
≥24	209 (59.7)	251 (71.7)		
**Physical activity**				
Low	17 (4.9)	12 (3.4)	1.811	0.404
Moderate	16 (4.6)	22 (6.3)		
High	317 (90.5)	316 (90.3)		
**Obesity**				
Normal	67 (19.1)	118 (33.7)	27.030	< 0.001
Overweight	170 (48.6)	167 (47.7)		
Obesity	113 (32.3)	65 (18.6)		
**Diabetes**				
No	320 (91.4)	330 (94.3)	1.620	0.203
Yes	30 (8.6)	20 (5.7)		
No	193 (55.1)	225 (64.3)	5.399	0.020
Yes	157 (44.9)	125 (35.7)		
**Occupational stress**				
No	123 (35.1)	157 (44.9)	6.482	0.011
Yes	227 (64.9)	193 (55.1)		
**Heat**				
No	123 (35.1)	191 (54.6)	24.134	< 0.001
Yes	227 (64.9)	159 (45.4)		
**Noise**				
No	144 (41.1)	189 (54.0)	11.877	0.001
Yes	206 (58.9)	161 (46.0)		
**Dust**				
No	153 (43.7)	203 (58.0)	13.049	< 0.001
Yes	197 (56.3)	147 (42.0)		
**CO**				
No	119 (34.0)	165 (47.1)	12.500	< 0.001
Yes	231 (66.0)	185 (52.9)		
**Shift work**				
Never	32 (9.1)	66 (18.9)	14.685	0.001
Former	56 (16.0)	59 (16.9)		
Now	262 (74.9)	225 (64.3)		
RTL^b^	1.05 (0.80, 1.35)	1.19 (0.89, 1.71)	−5.428	< 0.001

^a^Indicates that the mean ± standard deviation is used for description, and the independent samples t-test is used for group comparison. ^b^Indicates that the median and interquartile range is used for description, and the Wilcoxon rank-sum test is used for group comparison.

### 3.2 Construction of the OHFS

**Figure 1:**
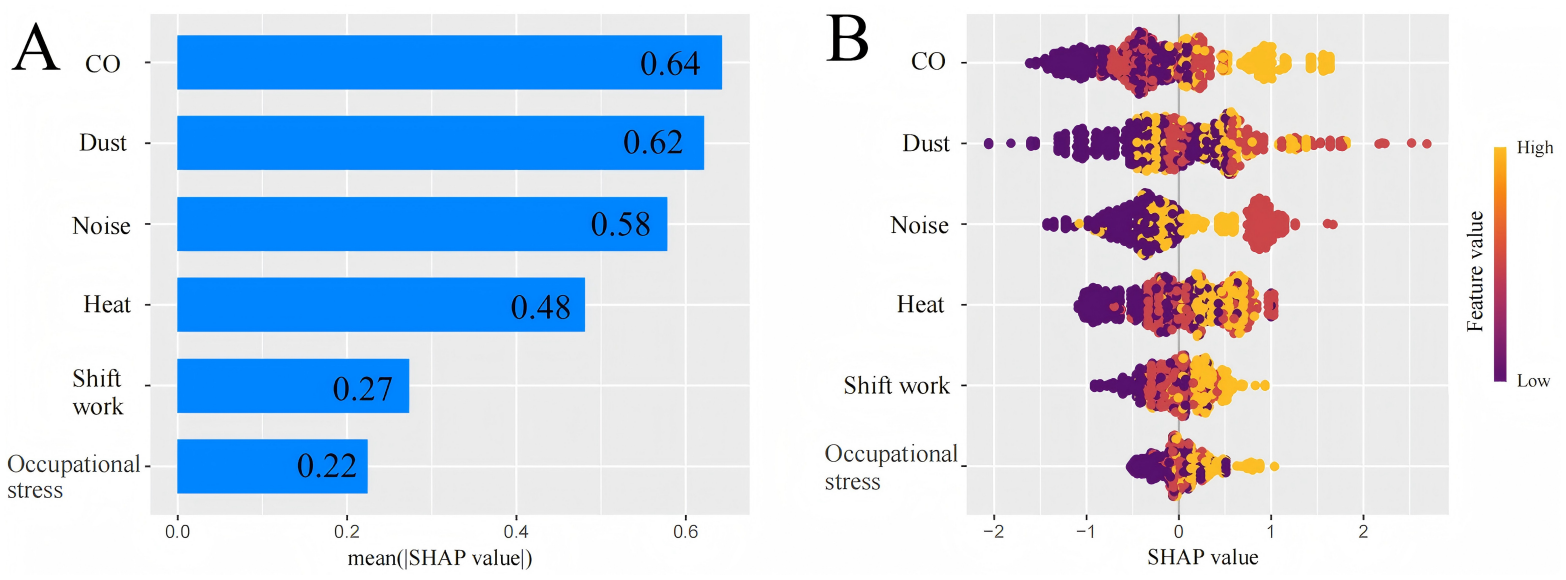
SHapley Additive Explanations (SHAP) for XGBoost variable importance ranking. **(A)** Sorting of relative importance of occupational hazardous factors. **(B)** Overall distribution of SHAP value of occupational hazardous factors.

Supplementary Figure S6 shows the relationships between various occupational hazardous factors and hypertension among male steel workers. Due to the small number of participants who never worked night shifts, the “never worked night shifts” group was combined with the “ever worked night shifts” group in subsequent analyses. Occupational hazards exposure was divided into low-years and high-years exposure based on the median of work years. After adjusting for confounding factors, exposure to heat (low duration: *OR* = 1.68, 95% *CI*: 1.12–2.52; high duration: *OR* = 2.08, 95% *CI*: 1.40–3.10), noise (low duration: *OR* = 1.74, 95% *CI*: 1.16–2.61; high duration: *OR* = 2.10, 95% *CI*: 1.40–3.15), dust (low duration: *OR* = 1.52, 95% *CI*: 1.01–2.27; high duration: *OR* = 2.49, 95% *CI*: 1.65–3.76), CO exposure (low duration: *OR* = 1.54, 95% *CI*: 1.03–2.29; high duration: *OR* = 2.22, 95% *CI*: 1.49–3.32), occupational stress (low duration: *OR* = 1.55, 95% *CI*: 1.06–2.28; high duration: *OR* = 1.69, 95% *CI*: 1.16–2.46), and now shift work (low duration: *OR* = 1.55, 95% *CI*: 1.06–2.27; high duration: *OR* = 1.86, 95% *CI*: 1.27–2.73) were associated with an increased risk of hypertension. The relationship between occupational hazards as binary variables and hypertension is shown in Supplementary Figure S7. To determine the importance of variables related to occupational hazardous factors, further analysis was conducted using the XGBoost model and SHapley Additive Explanations (SHAP). The specific ranking of variable importance is shown in Figure 1. The occupational hazardous factors were ranked by importance from highest to lowest as follows: CO, dust, noise, heat, shift work, and occupational stress. Their relative importance values were 0.64, 0.62, 0.58, 0.48, 0.27, and 0.22, respectively. Using occupational stress (0.22) as the unit of comparison, scores were assigned to each occupational hazardous factor. The scoring details are presented in Table 2.

**Table 2:** Scoring table for occupational hazardous factors among male steel workers.

**Occupational hazardous factors**	**Hazardous score**
CO	0 = No, 11.39 = Low duration exposure, 22.78 = High duration exposure
Dust	0 = No, 11.03 = Low duration exposure, 22.06 = High duration exposure
Noise	0 = No, 10.32 = Low duration exposure, 20.64 = High duration exposure
Heat	0 = No, 8.54 = Low duration exposure, 17.08 = High duration exposure
Shift work	0 = Never/Former, 4.81 = Low duration exposure, 9.61 = High duration exposure
Occupational stress	0 = No, 3.92 = Low duration exposure, 7.83 = High duration exposure

### 3.3 The relationship between OHFS and hypertension

The OHFS was divided into four groups based on quartiles: Q1 (< 24.74), Q2 (24.74~), Q3 (38.98~), and Q4 (≥56.58). OHFS (both continuous and categorical) was used as the independent variable, and the presence of hypertension as the dependent variable to analyze the relationship between OHFS and hypertension, as shown in Table 3. The results showed that the risk of hypertension among male steel workers in the higher OHFS groups (24.74~, 38.98~, and ≥56.58) was 1.81, 2.17, and 3.46 times higher than that in the lower OHFS group (< 24.74), respectively (24.74~: *OR* = 1.81, 95% *CI*: 1.14–2.86; 38.98~: *OR* = 2.17, 95% *CI*: 1.39–3.39; ≥56.58: *OR* = 3.46, 95% *CI*: 2.18–5.49). See Supplementary Table S4 for more details.

**Table 3:** The relationship between OHFS and hypertension.

**Variable**	**Model 1**		**Model 2**		**Model 3**	
	***OR*** **(95%** ***CI*****)**	***P***	***OR*** **(95%** ***CI*****)**	***P***	***OR*** **(95%** ***CI*****)**	***P***
OHFS (Continuous)	1.03 (1.02–1.04)	< 0.001	1.03 (1.02–1.04)	< 0.001	1.03 (1.02–1.04)	< 0.001
**OHFS (Categorical)**						
Q1 (< 24.74)	1.00 (Reference)		1.00 (Reference)		1.00 (Reference)	
Q2 (24.74~)	1.83 (1.18–2.84)	0.007	1.78 (1.14–2.79)	0.011	1.81 (1.14–2.86)	0.012
Q3 (38.98~)	2.11 (1.38–3.23)	0.001	2.03 (1.32–3.12)	0.001	2.17 (1.39–3.39)	0.001
Q4 (≥56.58)	3.24 (2.09–5.03)	< 0.001	3.06 (1.96–4.80)	< 0.001	3.46 (2.18–5.49)	< 0.001

Model 1: unadjusted. Model 2: adjusted for education level, smoking, drinking, and DASH score. Model 3: adjusted for obesity, diabetes, and dyslipidemia on the basis of Model 2.

### 3.4 The relationship between RTL and hypertension

Table 4 shows the relationship between RTL and hypertension. In the fully adjusted model, when RTL is treated as a continuous variable, shorter RTL is associated with a higher risk of hypertension (*OR* = 0.49, 95% *CI*: 0.36–0.68). When RTL is categorized, the risk of hypertension in the shorter RTL group was 1.45 times higher than that in the longer RTL group (*OR* = 1.45, 95% *CI*: 1.04–2.03). After standardizing RTL, for every 1 SD increase in RTL, the risk of hypertension decreases by 37%. The RCS plot showing the relationship between RTL and hypertension is presented in Supplementary Figure S8. See Supplementary Table S5 for more details.

**Table 4:** The relationship between RTL and hypertension.

**Variable**	**Model 1**		**Model 2**		**Model 3**	
	***OR*** **(95%** ***CI*****)**	***P***	***OR*** **(95%** ***CI*****)**	***P***	***OR*** **(95%** ***CI*****)**	***P***
RTL (continuous)	0.42 (0.32–0.56)	< 0.001	0.41 (0.30–0.55)	< 0.001	0.49 (0.36–0.68)	< 0.001
For each 1 SD increase in RTL	0.57 (0.48–0.69)	< 0.001	0.56 (0.46–0.68)	< 0.001	0.63 (0.52–0.78)	< 0.001
**RTL (categorize)**						
Long	1.00 (Reference)		1.00 (Reference)		1.00 (Reference)	
Short	1.65 (1.23–2.24)	0.001	1.70 (1.24–2.33)	0.001	1.45 (1.04–2.03)	0.029

Model 1: unadjusted. Model 2: adjusted for education level, smoking, drinking, DASH score, obesity, diabetes, and dyslipidemia. Model 3: further adjusted for Heat, Noise, Dust, CO, Shift work, and Occupational Stress based on Model 2. SD: 0.64.

### 3.5 The combined impact of OHFS and RTL on hypertension

After dividing the OHFS into groups based on the median and conducting joint effect analysis with RTL, the results in Table 5 show that individuals with shorter RTL and higher OHFS have the highest risk of hypertension (*OR* = 2.79, 95% *CI*: 1.83–4.25). Moreover, there is a multiplicative interaction between RTL and OHFS (*P* for interaction < 0.001), but no additive interaction. The joint effects of each occupational hazardous factors with RTL and their multiplicative and additive interactions are shown in Supplementary Table S6. The results showed that there are multiplicative interactions between heat, dust, shift work, and RTL.

**Table 5:** The combined impact of OHFS and RTL on hypertension.

**OHFS**	**RTL**	**β**	***Wald***	***OR* (95% *CI*)**	***P***	***P* for interaction**	**Additive interaction**
OHFS (Low)	RTL (Long)			1.00 (Reference)		< 0.001	RERI: 0.43 (−3.35–4.20) AP: 0.129 (−0.86–1.11) SI: 1.15 (0.87–1.43)
	RTL (Short)	0.51	4.95	1.66 (1.06–2.59)	0.026		
OHFS (High)	RTL (Long)	0.62	7.70	1.85 (1.99–2.86)	0.006		
	RTL (Short)	1.03	22.90	2.79 (1.83–4.25)	< 0.001		

The model was adjusted for education level, smoking, drinking, DASH score, obesity, diabetes, and dyslipidemia.

### 3.6 The relationship between OHFS and RTL

Table 6 explores the impact of the OHFS on RTL using quantile regression. The results show that when the OHFS is treated as a continuous variable, it has a significant impact across all quantiles of RTL (*P* < 0.05). When the OHFS is categorized, the Q3 and Q4 groups have a significant impact across all quantiles, while the Q2 group has a significant impact at the 50% and 90% quantiles (*P* < 0.05). The quantile regression results for each occupational hazardous factors and RTL are shown in Supplementary Table S7. Heat, noise, dust, and CO exposure have a significant impact across all quantiles of RTL (*P* < 0.05). High-duration exposure to occupational stress has a significant impact at the 10% and 50% quantiles (*P* < 0.05). No significant differences were found for shift work across any RTL quantiles. See Supplementary Table S8 for more details.

**Table 6:** Quantile regression analysis of the relationship between OHFS and RTL.

**OHFS**	**Q10**		**Q50**		**Q90**	
	*β **(95% CI)***	***P***	*β **(95% CI)***	***P***	*β **(95% CI)***	***P***
OHFS	−0.005 (−0.008, −0.003)	< 0.001	−0.007 (−0.009, −0.005)	< 0.001	−0.016 (−0.021, −0.011)	< 0.001
**OHFS (Reference: Q1)**						
OHFS (Q2: 24.74~)	−0.074 (−0.220, 0.073)	0.323	−0.130 (−0.260, −0.028)	0.048	−0.227 (−0.447, −0.006)	0.044
OHFS (Q3: 38.98~)	−0.121 (−0.237,−0.005)	0.042	−0.176 (−0.316, −0.036)	0.014	−0.252 (−0.483, −0.021)	0.032
OHFS (Q4: ≥56.58)	−0.194 (−0.320,−0.069)	0.002	−0.246 (−0.425, −0.066)	0.008	−0.349 (−0.573, −0.125)	0.002

The model was adjusted for education level, smoking, drinking, DASH score, obesity, diabetes, and dyslipidemia.

### 3.7 Mediation analysis of RTL

**Figure 2:**
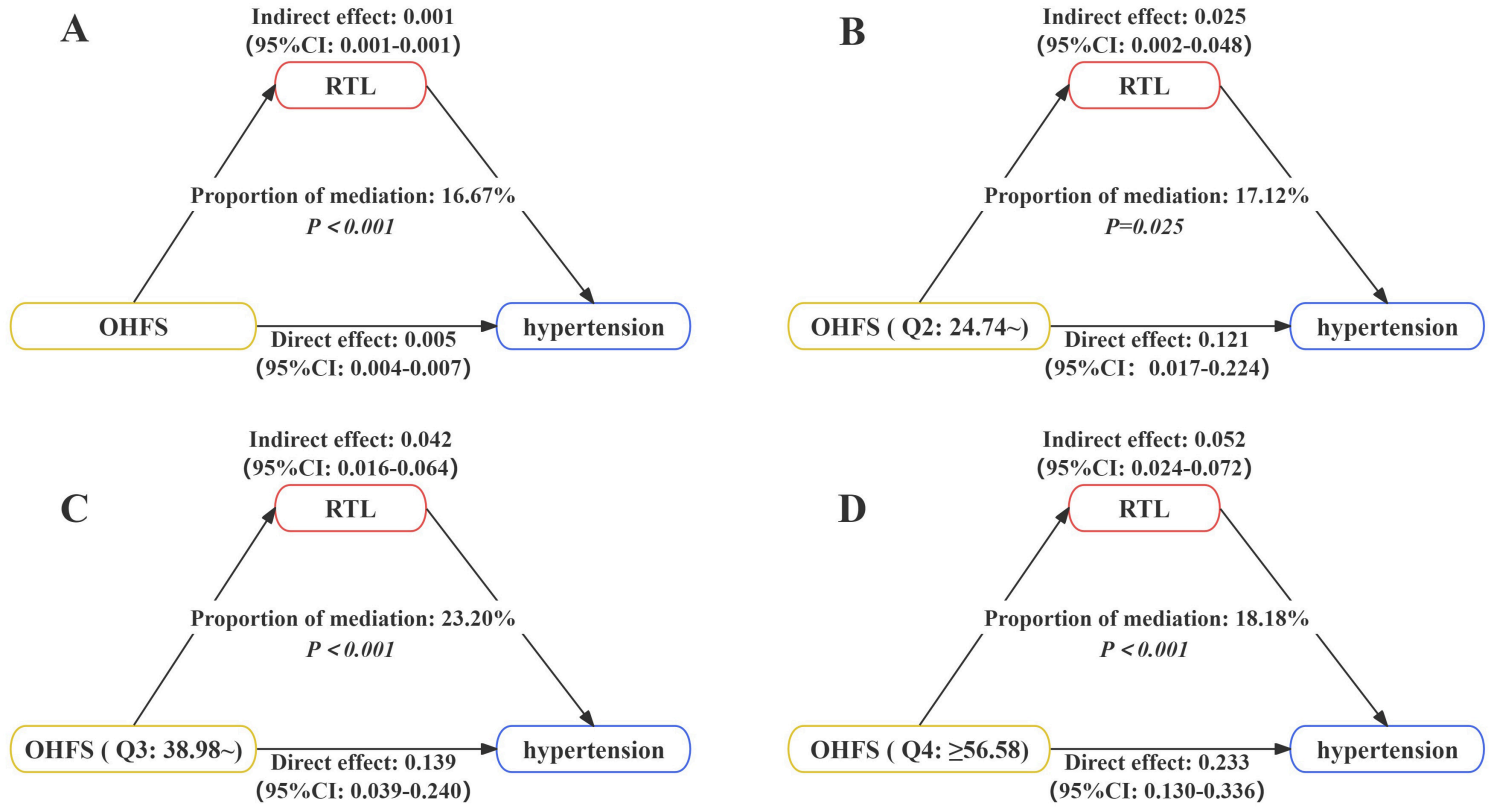
The mediating role of RTL in the relationship between OHFS and hypertension. The model was adjusted for education level, smoking, drinking, DASH score, obesity, diabetes, and dyslipidemia. **(A)** OHFS was analyzed as a continuous variable. **(B–D)** OHFS was analyzed as categorize variable.

The mediation analysis results show that after adjusting for confounding factors, RTL mediates the relationship between OHFS and hypertension. When OHFS is treated as a continuous variable, the mediation effect of RTL is 16.67%, as shown in Figure 2A. When OHFS is categorized, the mediation effects are 17.12%, 23.20%, and 18.18% for the respective groups, as shown in Figures 2B–D. The mediation effect decomposition table is presented in Supplementary Table S9. Additionally, Supplementary Figure S9 shows the mediation effect of RTL in the relationship between different durations of occupational hazard exposure and hypertension. RTL mediates the relationship between hypertension and heat (low duration: 13.10%; high duration: 18.01%), noise (low duration: 22.66%; high duration: 31.65%), dust (low duration: 20.72%; high duration: 26.29%), and CO (high duration: 18.33%). The mediation effect decomposition table is shown in Supplementary Table S10.

### 3.8 Stratified analysis

**Figure 3:**
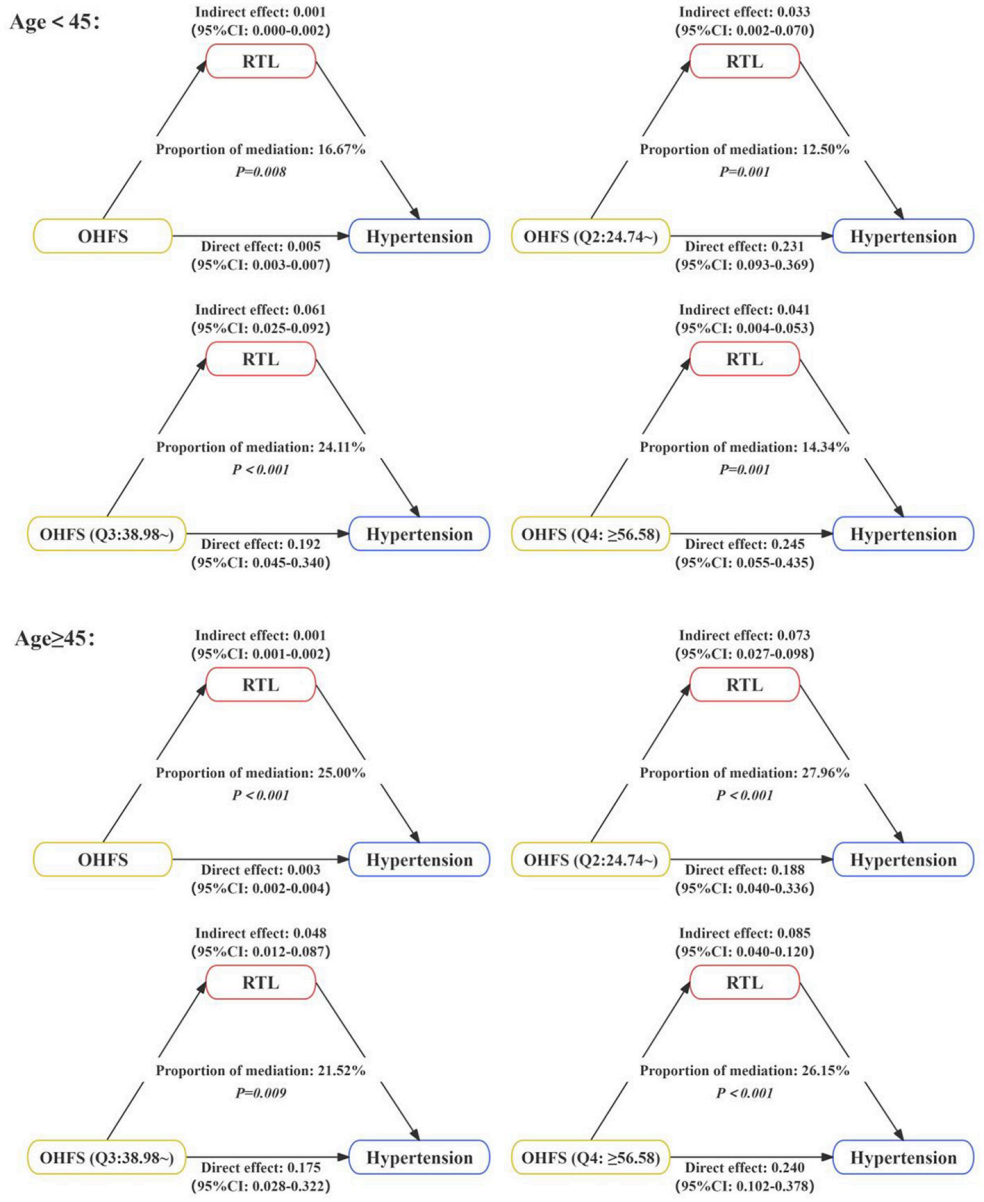
The mediating role of RTL in the relationship between OHFS and hypertension stratified by age. The model was adjusted for education level, smoking, drinking, DASH score, obesity, diabetes, and dyslipidemia.

Stratified by age, the mediating role of RTL in the relationship between OHFS and hypertension was explored, as shown in Figure 3. Among participants aged < 45 years, RTL mediated the relationship between OHFS and hypertension, with mediation effect proportions of 16.67% when OHFS was continuous, and 12.50%, 24.11%, and 14.34% when OHFS was categorical. Among participants aged ≥45 years, RTL still mediated this relationship, with mediation effect proportions of 25.00% when OHFS was continuous, and 27.96%, 21.52%, and 26.15% when OHFS was categorical. The effect decomposition tables are presented in Supplementary Table S11.

### 3.9 Sensitivity analysis

We conducted several sensitivity analyses. First, we treated occupational hazards as binary variables and analyzed the mediating role of RTL in their relationship with hypertension. The effect decomposition table is presented in Supplementary Table S12. Additionally, we stratified by age and analyzed the mediating role of RTL in the relationship between binary occupational hazards and hypertension. The results are shown in Supplementary Table S13. In both the < 45 years and ≥45 years age groups, RTL mediated the relationship between hypertension and high temperature, noise, dust, and CO. The mediating effects of RTL in the relationship between hypertension and high temperature and noise were larger in the ≥45 years group, at 20.00% and 37.86%, respectively, while the mediating effects in the relationship between hypertension and dust and CO were larger in the < 45 years group, at 28.47% and 25.18%, respectively. Furthermore, after excluding patients with coronary heart disease and atherosclerosis, we conducted mediation analyses, the results are shown in Supplementary Tables S14, S15. RTL mediated the relationship between OHFS and hypertension, as well as between high temperature, noise, dust, and hypertension. In addition, there are 58 new cases of hypertension in this study, and there are few new cases, so it is impossible to keep the new cases for analysis. Therefore, we excluded the new cases of hypertension for mediation analysis. See Supplementary Table S16 for detailed results. These sensitivity analyses indicate that the results of our study are robust.

## 4 Discussion

This study adopted a 1:1 matched case-control design. The results showed that occupational hazard factors (heat, noise, dust, CO, occupational stress, and shift work) may be risk factors for hypertension among male steel workers, with longer exposure durations showing a positive correlation with hypertension risk. Furthermore, by integrating multiple occupational hazards, we constructed an OHFS. It was found that higher scores may be associated with an increased risk of hypertension (24.74: *OR* = 1.81, 95% *CI*: 1.14–2.86; 38.98–56.57: *OR* = 2.17, 95% *CI*: 1.39–3.39; ≥56.58: *OR* = 3.46, 95% *CI:* 2.18–5.49). The shorter the relative RTL, the higher the risk of hypertension of male steel workers (*OR* = 0.49, 95% *CI*: 0.36–0.68). Additionally, higher OHFS was associated with shorter RTL. We also found that RTL partially mediated the relationship between OHFS and hypertension in male steel workers, with the mediation effect ranging from 17.12% to 23.20% across different score groups.

In 1959, Morris first proposed that occupational environments could also cause cardiovascular diseases, which has since attracted the attention of scholars both domestically and internationally (28). Hypertension, as an important component of cardiovascular diseases, has a much higher prevalence among steel workers than in the general population, reaching as high as 33.89% (6). Steel workers are exposed to numerous occupational hazards such as heat, noise, and dust during their work, which may lead to the occurrence and development of hypertension. Building on the research group's previous work, this study integrated multiple occupational hazards to construct an OHFS and found that a higher score is associated with an increased risk of hypertension. The research group has previously thoroughly explored the relationship between occupational hazards such as heat, noise, dust, CO, and shift work and the occurrence and development of hypertension, as well as the potential biological mechanisms involved (29–31). This study also found that occupational stress is associated with hypertension among steel workers. Ailing et al. (32) found that occupational stress is a risk factor for hypertension among oil field workers (*OR* = 1.243, 95% *CI*: 1.027–1.505, *P* = 0.025), and Gu (33) also found that occupational stress is associated with an increased risk of hypertension among Chinese petrochemical workers (*OR* = 1.50, 95% *CI*: 1.133–1.960). This may be because long-term exposure to occupational stress can lead to abnormal excitation of the sympathetic nervous system, which stimulates the hypothalamic-pituitary-adrenal axis, thereby promoting the accelerated secretion of adrenaline, noradrenaline, and other substances. This can lead to metabolic disorders and increased myocardial contractility, ultimately resulting in elevated blood pressure (34).

In addition, we found that RTL may be a protective factor against hypertension, with shorter telomeres associated with an increased risk of hypertension (*OR* = 0.49, 95% *CI*: 0.36–0.68). The RTL in the hypertension group among steel workers was 0.14 T/S units shorter than that in the control group. Several studies on occupational populations have also found that shortened telomere length is associated with an increased risk of hypertension (35, 36). Research has shown that the gradual shortening of telomeres is a key mechanism of cellular aging, and accelerated telomere shortening is often considered a common feature of age-related diseases (37). As an effective indicator of vascular aging and related diseases, shortened telomere length may accelerate the aging of vascular endothelial cells, increasing the risk of vascular damage and thereby promoting the occurrence and development of hypertension (38). Animal experiments have found that extremely short telomeres in telomere-deficient mice lead to elevated blood pressure, which is associated with increased levels of circulating endothelin-1 and increased production of reactive oxygen species, indicating that telomere attributes may play an important role in the etiology of hypertension (39).

In addition to genetic factors, the impact of exogenous harmful factors on telomere length attrition has garnered increasing attention. We found that among steel workers, the distribution of RTL across quantiles differs significantly by OHFS and exposure to high temperature, noise, dust, and CO, which is consistent with the findings of a retrospective cross-sectional study by Ouyang et al. (40) using data from the National Health and Nutrition Examination Survey (NHANES) that showed an association between lead and cadmium exposure and shortened telomere length, though with a larger effect size, likely due to higher exposure levels among steel workers. Dust and CO may stimulate the production of reactive oxygen species (ROS) in the body, which directly attack telomere DNA and induce single-strand breaks. Noise exposure, through chronic activation of the sympathetic-adrenal axis, increases oxidative stress and inhibits telomerase activity, leading to shortened telomere length. Heat exposure, associated with an imbalance in heat shock protein homeostasis, can also accelerate telomere attrition. However, no association was observed between occupational stress and shift work and telomere length. Although few studies have directly examined the impact of occupational stress and shift work on telomere length, research has shown that psychological factors (41) and circadian rhythm disruption (42) are associated with shortened telomere length. However, our research has not found the relationship between occupational stress and shift work and telomere length, which needs further study to confirm their relationship.

Hypertension is influenced by environmental factors and genetic factors. Environmental factors can lead to changes in genetic factors, thus affecting the disease, and environmental factors and genetic factors can work together to affect the disease. Therefore, the study examined the two relationships between OHFS and RTL in influencing hypertension, with RTL playing a partial mediating role in the association between OHFS and hypertension. Long-term exposure to various occupational hazards is linked to an increased risk of hypertension, and RTL partially mediates this relationship. The mediating effect suggests that OHFS may affect hypertension risk through two pathways: one is the direct pathway, where OHFS directly influences hypertension risk, and the other is the indirect pathway, where OHFS may accelerate RTL shortening, thereby elevating hypertension risk. Additionally, a multiplicative interaction was observed between OHFS and RTL, indicating that their combined effect on hypertension risk is not simply additive. The state of RTL may modify or alter the strength of OHFS's impact on hypertension. This implies that individuals with shorter RTL may face a higher risk of hypertension when exposed to the same occupational hazards. This interaction highlights the synergistic and amplifying effects of biological susceptibility and environmental exposure in disease development. Our findings provide a theoretical basis for interventions aimed at reducing occupational exposure or exploring protective agents (such as antioxidants), which may help mitigate telomere shortening and reduce the risk of hypertension.

In summary, this study, focusing on steel workers, systematically explored the relationship between comprehensive exposure to multiple occupational hazards and RTL with hypertension, as well as the interaction between them. It also assessed the potential mediating role of RTL in the association between comprehensive occupational hazard exposure and hypertension. This not only provides a new explanatory framework for occupational cardiovascular damage but also positions telomeres as molecular targets for early intervention, shifting the focus from “disease management” to “health maintenance.” It offers a scientific basis for developing precision occupational health strategies based on telomere biomarkers.

However, this study still has certain limitations. Firstly, the case-control study design makes it difficult to determine the temporal sequence among “exposure, telomere shortening, and hypertension.” Hypertension itself can lead to physiological and oxidative stress, which in turn accelerates telomere wear. However, due to the limited sample size in this study, it is challenging to conduct sensitivity analyses using only newly diagnosed hypertension cases. Therefore, the study cannot fully rule out reverse causality bias. Future prospective cohort studies or nested case-control studies are needed to explore their causal relationships. Second, although the study meets the minimum sample size requirement for a 1:1 matched case-control study, the overall sample size is still relatively small, and larger samples are needed for confirmatory studies. Despite the use of matching, multivariate, and stratified methods to control for confounding as much as possible, there may still be unmeasured or imprecisely measured confounders. For example, due to sample size issues, we did not exclude individuals with diabetes or dyslipidemia but instead controlled them as confounding factors. Due to limited data, exposure was not calculated as cumulative exposure but was treated as a binary variable. Therefore, in future studies, exposure data should be improved, quantitative calculations should be performed, and mixed-effects models should be added. Finally, we used qPCR to measure telomere length. Although strict quality control measures were implemented, such as DNA quality, blinded testing, and primer efficiency, measurement errors may still exist due to instrumentation. Therefore, more in-depth and rigorous studies are needed in the future to further explore this area. Additionally, although we initially planned a 1:1 matching between case and control groups based on age and gender during the study design phase, only male steel workers were included in the case group. As a result, the matched study population was limited to male steel workers, which constrains the generalizability of our findings. Future studies should increase the sample size to better represent the overall steel worker population.

## 5 Conclusion

This study employed the XGBoost and SHAP analytical models to develop a comprehensive OHFS incorporating multiple factors such as heat, noise, dust, carbon monoxide exposure, shift work, and occupational stress. Among male steel workers, the higher the OHFS, the higher the risk of hypertension. In addition, in this population, the shorter the RTL, the higher the risk of hypertension, and RTL partially mediated the relationship between OHFS and hypertension. Collectively, these insights offer a novel perspective for understanding occupation-related cardiovascular impairment and support the further investigation of telomere length as a molecular biomarker for early health intervention.

## Data Availability

The raw data supporting the conclusions of this article will be made available by the authors, without undue reservation.
