# Supplementary material for: Association between comprehensive exposure to multiple occupational hazardous factors and telomere length with hypertension in male steel workers: a case-control study
Source: Front Public Health. 2026 Jan 30;14:1757027. doi: 10.3389/fpubh.2026.1757027 (PMC12901486; doi:10.3389/fpubh.2026.1757027)
Supplement: Supplementary file 1 [file Data_Sheet_1.pdf]

Supplementary Material

1 Supplementary Methods

1.1 Classification of covariates and their relevant definitions

1) Age: Continuous, categorized (<45 years, ≥45 years).

2) Education level: Low level (junior high school and below), Medium level (high school/ ocational school/technical school), High level (associate degree and above).

3) Marital status: Unmarried, Married, Other (remarried, divorced, widowed).

4) Income: Per capita monthly family income (<1500, 1500-3000, ≥3000).

5) Smoking: Subjects who smoke at least one cigarette per day for more than six months were defined as current smokers; those who had quit smoking for more than one year were defined as former smokers. Categorized as Never smoked, Former smoker, and Now smoker.

6) Drinking: Subjects who drink at least once a week for more than six months were defined as current drinkers; those who had quit drinking for more than one year were defined as former drinkers. Categorized as Never drinker, Former drinker, and Now drinker.

7) Diet (1): This study investigated the dietary habits of the subjects, including the frequency of consumption of eight types of food: whole grains, fresh vegetables, nuts and legumes, dairy and dairy products, sugary drinks, red meat and processed or pickled meat products, and sodium intake. The DASH (Dietary Approaches to Stop Hypertension) dietary index score was calculated based on the DASH dietary pattern proposed by Professor Fung et al. Each type of food was divided into five categories based on weekly intake frequency, and scores of 1-5 were assigned according to the frequency. Higher intake frequencies of whole grains, fresh fruits, fresh vegetables, nuts and legumes, and dairy and dairy products were assigned higher scores (Q5 = 5 points); lower intake frequencies of sugary drinks, red meat and processed meat products, and sodium were assigned higher scores (Q1 = 5 points). The total score of the dietary index ranged from 8 to 40 points and was categorized as (<24, ≥24).

8) Physical activity (2): The International Physical Activity Questionnaire (long version) (IPAQ), translated and revised by Chinese scholar Ningning Qu, was used to investigate the physical activity of the subjects. The questionnaire surveyed the intensity, duration, and frequency of physical activity related to work, transportation, daily household chores, and leisure exercise over the past week, with a total of 27 questions. According to the IPAQ scoring method, the physical activity level and its metabolic equivalent of task (MET) were assigned values, converting each individual's weekly activity into MET-minutes. Subjects who engaged in less than 600

MET-minutes of activity per week were classified into the low physical activity group, 600 to 3000 MET-minutes into the moderate physical activity group, and more than 3000 MET-minutes into the high physical activity group.

9) Obesity (3): According to the Chinese Guidelines for the Prevention and Control of Overweight and Obesity in Adults, BMI was categorized into four groups. BMI ≥28.0 kg/m² was defined as obese, 24.0 kg/m² ≤ BMI < 28.0 kg/m² as overweight, 18.5 kg/m² ≤ BMI < 24.0 kg/m² as normal weight, and BMI < 18.5 kg/m² as underweight. BMI = Weight (kg) / Height (m)². Since there were only five individuals classified as underweight in the study, they were combined with the normal weight group.

10) Diabetes (4): According to the "China Type 2 Diabetes Prevention and Treatment Guidelines (2024 Edition)," diabetes is defined as having a 2h-PG ≥11.1 mmol/L, or FBG ≥7.0 mmol/L, or HbA1c ≥6.5%, or having a history of diabetes and currently undergoing treatment.

11) Dyslipidemia (5): According to the "China Lipid Management Guidelines (Primary Care Edition 2024)," dyslipidemia is defined as having at least one of the following criteria: serum total cholesterol ≥6.2 mmol/L (240 mg/dl), triglycerides ≥2.3 mmol/L (200 mg/dl), low-density lipoprotein cholesterol ≥4.1 mmol/L (160 mg/dl), or non-high-density lipoprotein cholesterol ≥4.9 mmol/L (190 mg/dl). If the study subject is taking relevant medications, they are also defined as having dyslipidemia, even if they do not meet the above criteria.

1. Fung TT, Chiuve SE, McCullough ML, Rexrode KM, Logroscino G, Hu FB. Adherence to a DASH-style diet and risk of coronary heart disease and stroke in women. *Arch Intern Med*(2008) 168:713-20. doi:10.1001/archinte.168.7.713

2. Qu NN, Li KJ. Study on the reliability and validity of international physical activity questionnaire (Chinese vision, IPAQ). *Zhonghua Liu Xing Bing Xue Za Zhi* (2004) 25:265-268. doi:10.3760/j.issn:0254-6450.2004.03.021

3. National Health and Family Planning Commission of the People's Republic of China. Criteria of weight for adults: WS/T 428-2013[S]. Beijing: Standards Press of China, 2013.

4. Chinese Diabetes Society. Guideline for the prevention and treatment of diabetes mellitus in China (2024 edition). *Chin J Diabetes Mellit* (2025) 17:16-139.

5. Joint Committee on the Chinese Guidelines for Lipid Management. Chinese guideline for lipid management (primary care version 2024). *Chin Gen Pract* (2024) 27:2429-2436. doi:10.12114/j.issn.1007-9572.2024.0005

2 Supplementary Figures and Tables

2.1 Supplementary Figures

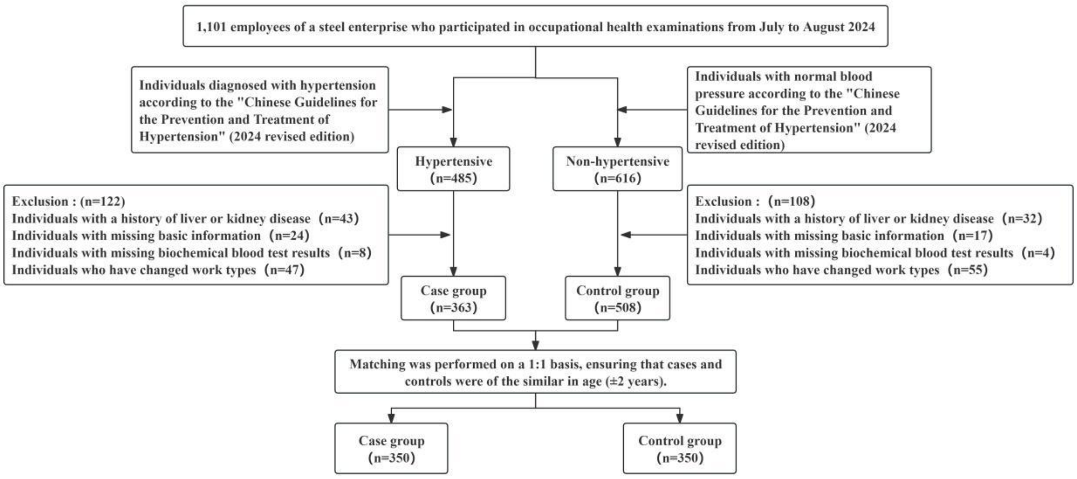

Figure S1 Flowchart of the selection process for study subjects

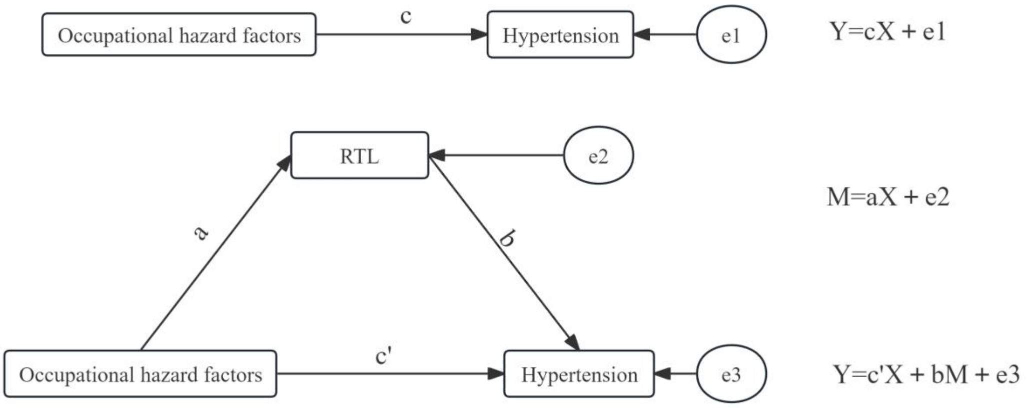

Figure S2 Mediation Effect Model Analysis Diagram

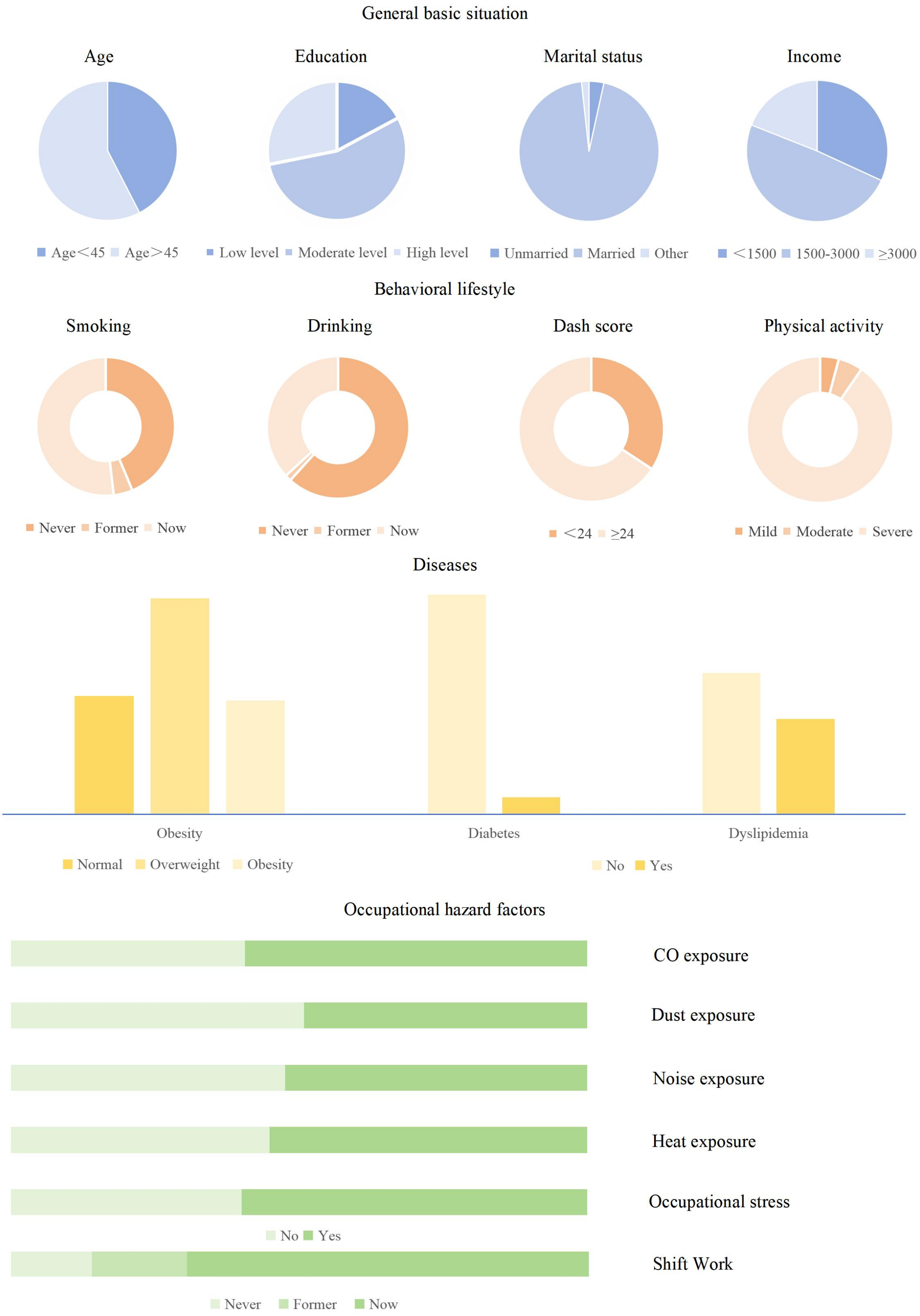

Figure S3 Distribution of basic characteristics in the total population

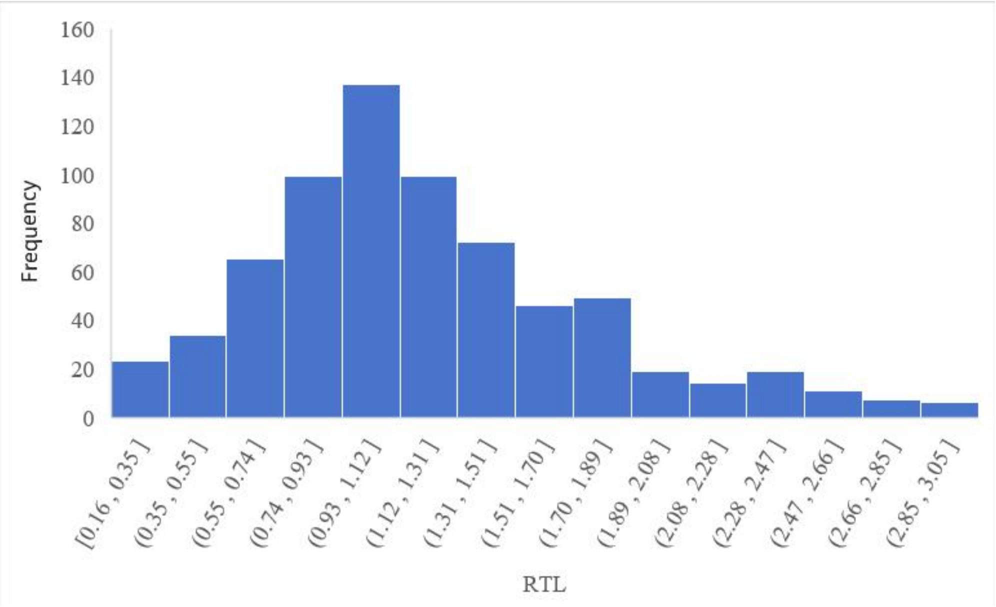

Figure S4 Distribution of RTL in the total population

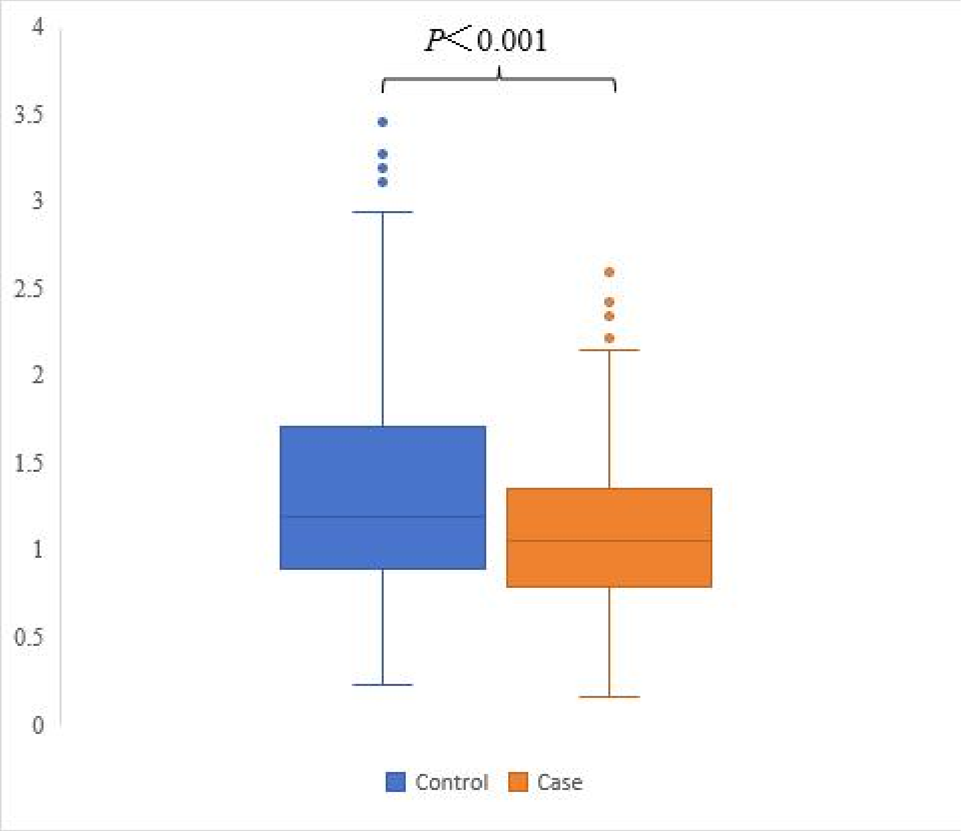

Figure S5 Box plot of RTL in the case group and the control group.Box plot explanation: The upper horizontal line of the box represents the 75th percentile; the lower horizontal line of the box represents the 25th percentile; the horizontal line inside the box represents the median; the upper horizontal line outside the box represents the maximum value excluding outliers; the lower horizontal line outside the box represents the minimum value excluding outliers; the dots represent outliers. $P < 0.05$ indicates that the difference between the two groups is statistically significant.

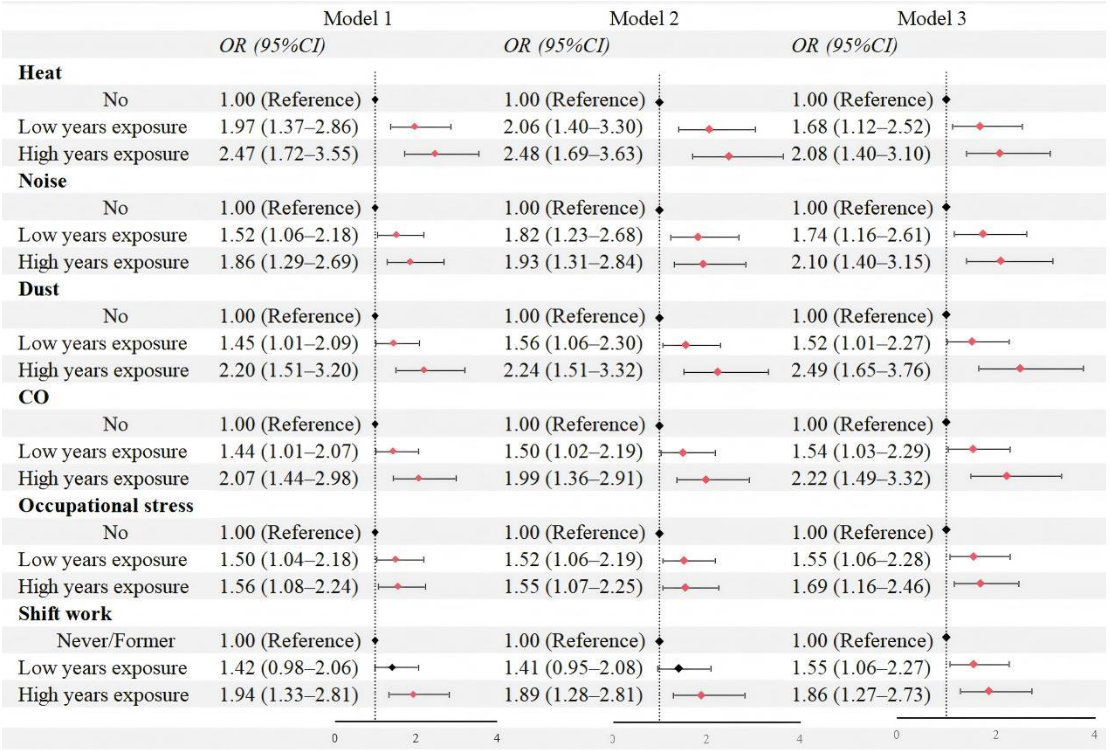

Figure S6 The relationship between different occupational hazardous factors exposure duration and hypertension among steel workers. Model 1: Unadjusted. Model 2: Adjusted for education level, smoking, drinking, and DASH score. Model 3: Further adjusted for obesity, diabetes, and dyslipidemia based on Model 2. Red: $P<0.05$.

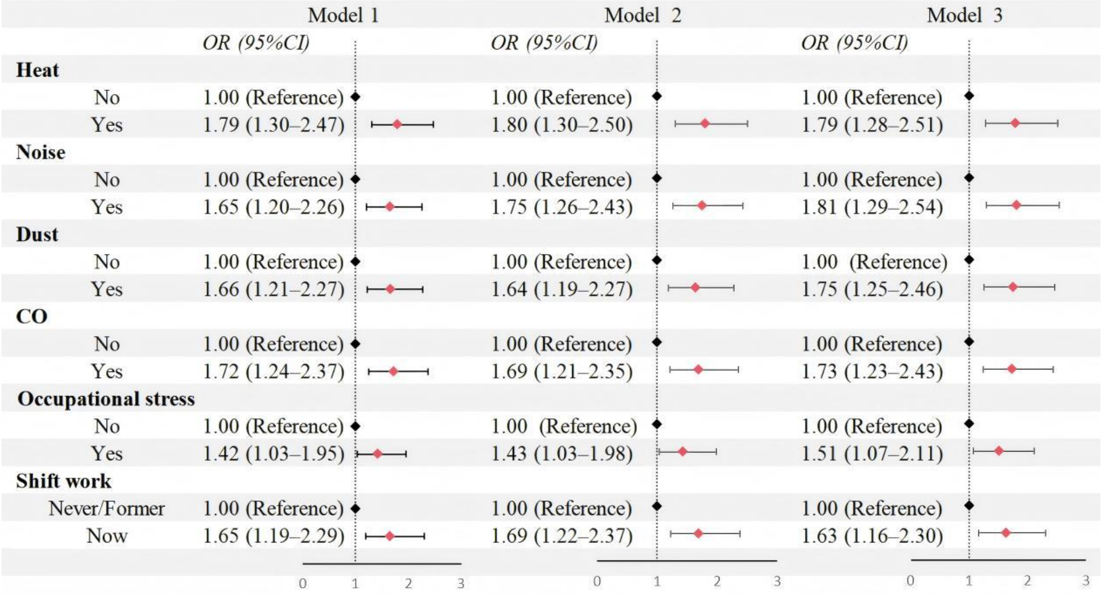

Figure S7 The relationship between occupational hazardous factors and hypertension.Model 1: Unadjusted. Model 2: Adjusted for education level, smoking, drinking, and DASH score. Model 3: Further adjusted for obesity, diabetes, and dyslipidemia based on Model 2. Red: $P<0.05$.

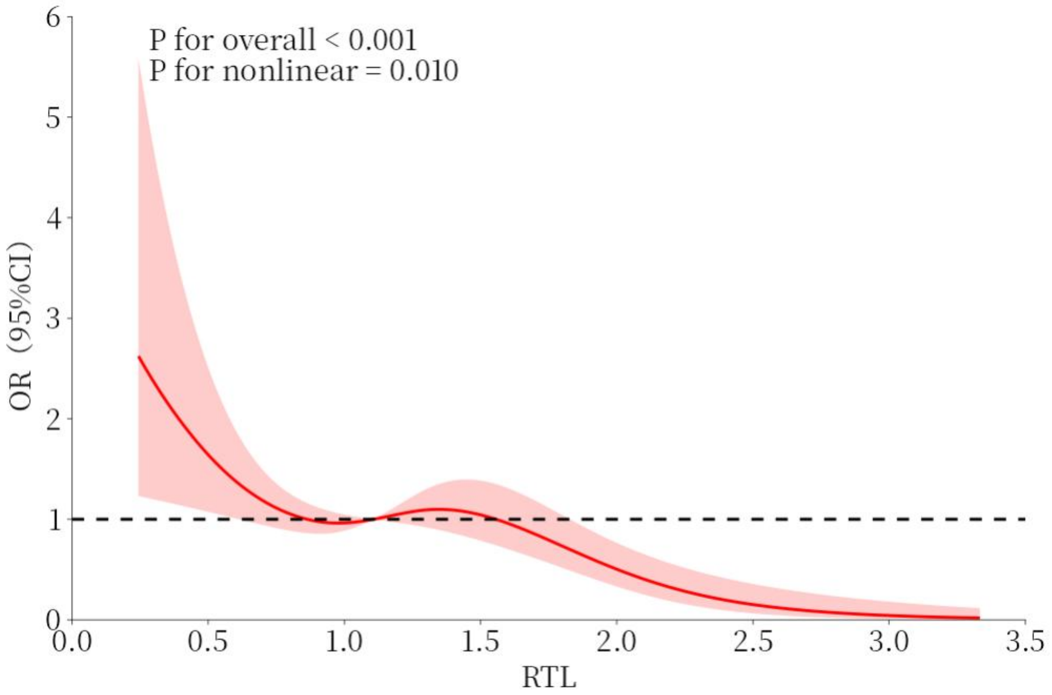

Figure S8 Restricted cubic spline plot showing the relationship between RTL and hypertension.The model was adjusted for education level, smoking, drinking, DASH score, obesity, diabetes, dyslipidemia, Heat, Noise, Dust, CO, Shift work, and Occupational Stress.

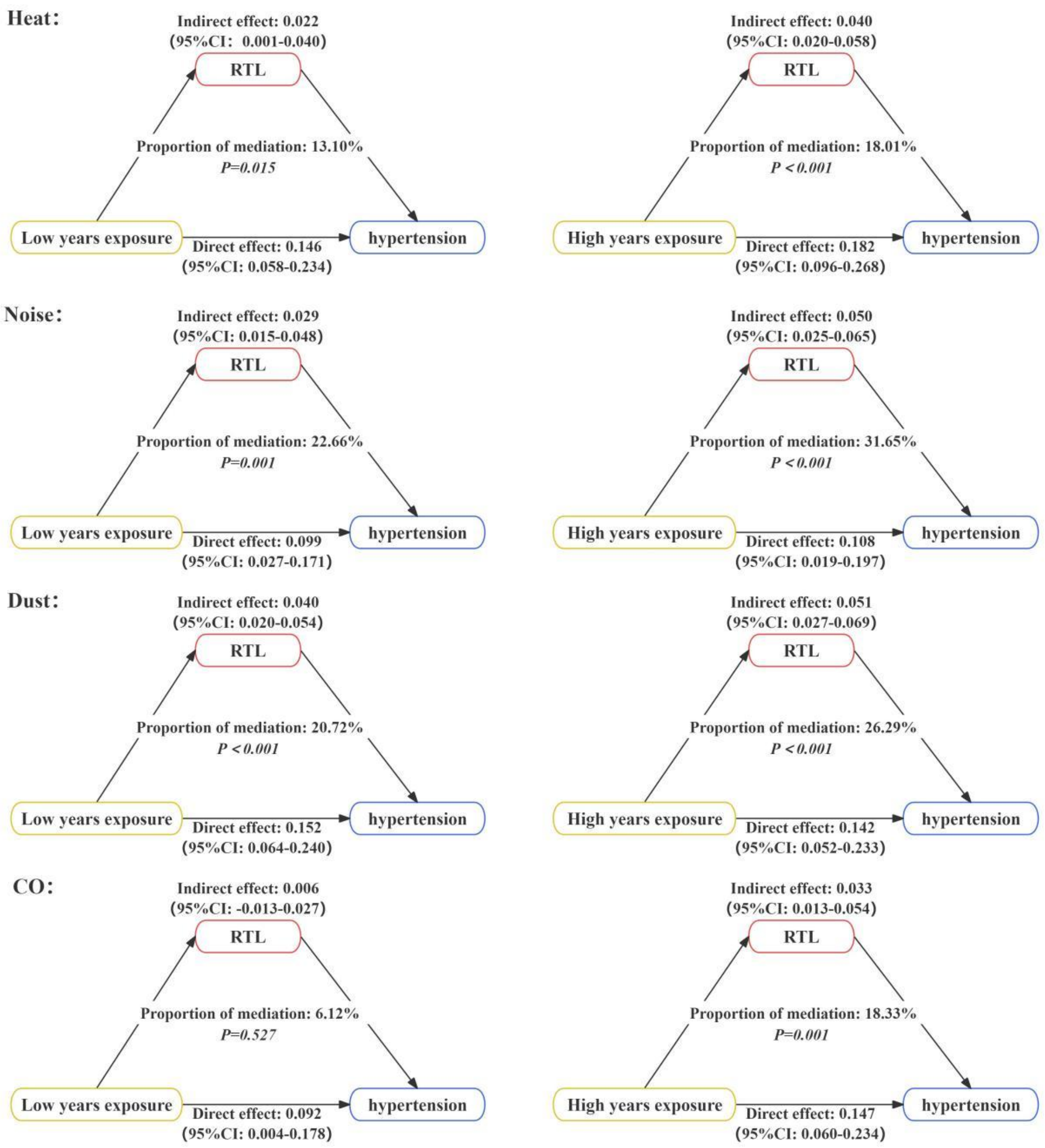

Figure S9 The mediating role of RTL in the relationship between different durations of occupational hazardous factors exposure and hypertension.The model was adjusted for education level, smoking, drinking, DASH score, obesity, diabetes, dyslipidemia, and other occupational hazards apart from the factors under study.

2.2 Supplementary Tables

Table S1 Telomere and 36B4 primer sequence information

Primer name	Sequence (5'-3')	nucleobase	GC(%)	Molecular weight
Tel F	CGG TTT GTT TGG GTT TGG GTT TGG GTT TGG GTT TGG GTT	39	48.7	12237.0
Tel R	GGC TTG CCT TAC CCT TAC CCT TAC CCT TAC CCT TAC CCT	39	53.8	11651.6
36B4 F	CAG CAA GTG GGA AGG TGT AAT CC	23	52.1	7137.7
36B4 R	CCC ATT CTA TCA TCA ACG GGT ACA A	25	44	7570.0

Tel: Telomere; F: Forward primer; R: Reverse primer

Table S2 PCR reaction system components

Component	Final concentration	Volume/μL
Nuclease-Free Water		6.8
Tel/36B4 F (10μM)	0.3 μM	0.6
Tel/36B4 R (10μM)	0.3 μM	0.6
DNA template (20 ng/μL)	2 ng/μL	2
GoTaq qPCR Master Mix, 2X	1X	10
Total Volume		20

Tel: Telomere; F: Forward primer; R: Reverse primer

Table S3 Settings for the PCR reaction program

Cycle	Stage	Temperature°C	Time	Fluorescence signal acquisition
1	Predenaturation	95	10min	No
40	Denaturation	95	15sec	No
	Annealing/Extension	60	1min	Yes
	Dissolution curve analysis	60–95		

Table S4 The Relationship between Occupational Hazard Factor Score (OHFS) and Hypertension in Fully Adjusted Model

Variable	*β*	Wald	OR (95% CI)	P
OHFS (Categorical)				
Q1 (<24.74)	1.00 (Reference)		1.00 (Reference)	
Q2 (24.74~)	0.59	6.38	1.81 (1.14-2.86)	**0.012**
Q3 (38.98~)	0.78	11.73	2.17 (1.39-3.39)	**0.001**
Q4 (≥56.58)	1.24	27.56	3.46 (2.18-5.49)	**<0.001**
Education				
Low	1.00 (Reference)		1.00 (Reference)	
Medium	-0.39	2.98	0.68 (0.43-1.06)	0.085
High	-0.36	2.05	0.70 (0.42-1.14)	0.152
Smoking				
Never	1.00 (Reference)		1.00 (Reference)	
Former	0.62	2.24	1.86 (0.83-4.20)	0.134
Now	0.23	1.78	1.26 (0.90-1.75)	0.182
Drinking				
Never	1.00 (Reference)		1.00 (Reference)	
Former	0.14	0.04	1.27 (0.24-3.24)	0.838
Now	0.39	5.22	1.48 (1.06-2.08)	0.022
DASH Score	-0.01	0.34	0.99 (0.96-1.02)	0.56
Obesity				
Normal	1.00 (Reference)		1.00 (Reference)	
Overweight	0.62	9.79	1.85 (1.26-2.72)	0.002
Obesity	1.19	26.36	3.28 (2.09-5.17)	<0.001

Diabetes				
No	1.00 (Reference)		1.00 (Reference)	
Yes	0.22	0.46	1.24 (0.66-2.33)	0.496
Dyslipidemia				
No	1.00 (Reference)		1.00 (Reference)	
Yes	0.25	2.30	1.29 (0.93-1.79)	0.129

Table S5 The Relationship between RTL and hypertension in Fully Adjusted Model

Variable	*β*	Wald	*OR (95% CI)*	*P*
RTL (categorize)				
Long	1.00 (Reference)		1.00 (Reference)	
Short	0.37	4.82	1.45 (1.04-2.03)	**0.029**
Education				
Low	1.00 (Reference)		1.00 (Reference)	
Medium	-0.62	6.76	0.54 (0.34-0.86)	0.009
High	-0.61	5.29	0.55 (0.33-0.91)	0.021
Smoking				
Never				
Former	0.47	1.14	1.59 (0.68-3.75)	0.286
Now	0.14	0.62	1.15 (0.81-1.64)	0.431
Drinking				
Never	1.00 (Reference)		1.00 (Reference)	
Former	-0.29	0.17	0.74 (0.19-2.97)	0.674
Now	0.47	6.87	1.60 (1.13-2.28)	0.009
DASH Score	-0.01	0.04	0.99 (0.96-1.03)	0.843
Obesity				
Normal	1.00 (Reference)		1.00 (Reference)	
Overweight	0.67	10.52	1.96 (1.30-2.94)	0.001
Obesity	1.17	23.31	3.21 (2.00-5.16)	<0.001
Diabetes				
No	1.00 (Reference)		1.00 (Reference)	
Yes	0.52	2.52	1.68 (0.89-3.20)	0.112
Dyslipidemia				
No	1.00 (Reference)		1.00 (Reference)	
Yes	0.22	1.59	1.25 (0.89-1.75)	0.207
Heat				
No	1.00 (Reference)		1.00 (Reference)	
Yes	0.58	11.51	1.79 (1.27-2.52)	0.001
Noise				
No	1.00 (Reference)		1.00 (Reference)	
Yes	0.57	10.72	1.76 (1.26-2.47)	0.001
Dust				
No	1.00 (Reference)		1.00 (Reference)	

Yes	0.5	8.46	1.66 (1.18-2.33)	0.004
CO				
No	1.00 (Reference)		1.00 (Reference)	
Yes	0.54	9.82	1.72 (1.23-2.42)	0.002
Occupational stress				
No	1.00 (Reference)		1.00 (Reference)	
Yes	0.4	5.27	1.49 (1.06-2.08)	0.022
Shift work				
Never	1.00 (Reference)		1.00 (Reference)	
Former	0.71	4.99	2.03 (1.09-3.78)	0.025
Now	0.79	9.54	2.20 (1.34-3.64)	0.002

Table S6 The Combined Impact of Occupational Hazardous Factors and RTL on Hypertension

RTL	Occupational Hazards	*OR* (95% *CI*)	*P*	*P* for interaction	Additive Interaction
	Heat			0.008	
RTL (Long)	No	1.00 (Reference)			RERI: 0.55 (0.19-0.91)
	Yes	2.13 (1.37-3.32)	0.001		AP: 0.97 (0.95-0.99)
RTL (Short)	No	1.74 (1.08-2.78)	0.022		SI: 2.44 (0.56-10.66)
	Yes	2.19 (1.41-3.41)	0.001		
	Noise			0.326	
RTL (Long)	No	1.00 (Reference)			RERI: 0.19 (-0.10-0.47)
	Yes	1.81 (1.16-2.85)	0.010		AP: 0.25 (-0.03-0.54)
RTL (Short)	No	1.64 (1.04-2.60)	0.035		SI: 1.33 (-0.49-3.16)
	Yes	2.27 (1.48-3.49)	<0.001		
	Dust			0.001	
RTL (Long)	No	1.00 (Reference)			RERI: 0.85 (0.40-1.29)
	Yes	1.59 (1.03-2.48)	0.038		AP: 0.74 (0.42-1.05)
RTL (Short)	No	1.58 (1.03-2.44)	0.036		SI: 3.79 (-4.04-11.63)
	Yes	1.79 (1.14-2.82)	0.011		
	CO			0.208	
RTL (Long)	No	1.00 (Reference)			RERI: 0.25 (-0.01-0.51)
	Yes	2.09 (1.32-3.32)	0.002		AP: 0.37 (0.12-0.61)
RTL (Short)	No	1.83 (1.11-3.02)	0.019		SI: 1.58 (-1.29-4.44)
	Yes	2.53 (1.60-4.00)	<0.001		
	Occupational Stress			0.518	
RTL (Long)	No	1.00 (Reference)			RERI: 0.09 (-0.12-0.30)
	Yes	1.49 (0.96-2.29)	0.073		AP: 0.13 (-0.13-0.39)
RTL (Short)	No	1.22 (0.74-1.99)	0.442		SI: 1.15 (-0.30-2.60)
	Yes	1.65 (1.04-2.62)	0.032		
	Shift work			0.013	RERI: 0.46 (0.23-0.69)
RTL (Long)	Never/Former	1.00 (Reference)			AP: 2.27 (0.83-3.71)
	Now	2.86 (1.62-5.05)	<0.001		SI: 0.68 (0.39-1.17)

RTL (Short)	Never/Former	2.52 (1.53-4.14)	<0.001
	Now	3.44 (2.08-5.68)	<0.001

The model was adjusted for education level, smoking, drinking, DASH score, obesity, diabetes, and dyslipidemia.

Table S7 Quantile Regression Analysis of the Relationship between Occupational Hazardous Factors and RTL

Occupational Hazards	Q10		Q50		Q90	
	β	*P*	*β*	*P*	*β*	*P*
Heat (Reference: No)						
Low years exposure	-0.127	0.039	-0.126	0.013	-0.275	0.002
High years exposure	-0.219	<0.001	-0.277	0.005	-0.391	<0.001
Noise (Reference: No)						
Low years exposure	-0.182	<0.001	-0.230	<0.001	-0.251	<0.001
High years exposure	-0.223	<0.001	-0.263	<0.001	-0.349	<0.001
Dust (Reference: No)						
Low years exposure	-0.183	0.007	-0.193	<0.001	-0.279	<0.001
High years exposure	-0.192	<0.001	-0.225	<0.001	-0.368	0.010
CO (Reference: No)						
Low years exposure	-0.123	0.049	-0.127	0.018	-0.202	0.004
High years exposure	-0.143	0.021	-0.149	0.009	-0.354	0.002
Shift work (Reference: Never/Former)						
Low years exposure	-0.028	0.642	0.052	0.374	-0.129	0.19
High years exposure	-0.032	0.526	0.116	0.350	-0.147	0.137
Occupational Stress (Reference: No)						
Low years exposure	-0.118	0.067	-0.088	0.317	-0.063	0.250
High years exposure	-0.139	0.028	-0.121	0.016	-0.150	0.242

The model was adjusted for education level, smoking, drinking, DASH score, obesity, diabetes, and dyslipidemia.

Table S8 Quantile Regression Analysis of the Relationship between OHFS and RTL in Fully Adjusted Model.

Variable	Q10		Q50		Q90	
	β (95%CI)	*P*	*β (95%CI)*	*P*	*β (95%CI)*	*P*
OHFS (Reference：Q1)						
OHFS (Q2:24.74~)	-0.074 (-0.220, 0.073)	0.323	-0.130 (-0.260, -0.028)	0.048	-0.227 (-0.447, -0.006)	0.044
OHFS (Q3:38.98~)	-0.121 (-0.237, -0.005)	0.042	-0.176 (-0.316, -0.036)	0.014	-0.252 (-0.483, -0.021)	0.032
OHFS (Q4:≥56.58)	-0.194 (-0.320, -0.069)	0.002	-0.246 (-0.425, -0.066)	0.008	-0.349 (-0.573, -0.125)	0.002
Education (Reference：Low)						
Low						
Medium	0.122 (0.004, 0.241)	0.043	-0.021 (-0.133, 0.090)	0.71	0.039 (-0.154, 0.232)	0.694
High	0.001 (-0.142, 0.145)	0.986	0.021 (-0.104, 0.146)	0.745	0.003 (-0.210, 0.217)	0.976
Smoking (Reference：Never)						
Never						

Former	-0.206 (-0.431, 0.019)	0.073	-0.114 (-0.318, 0.090)	0.273	-0.143 (-0.329, 0.043)	0.131
Now	-0.223 (-0.508, 0.062)	0.125	-0.163 (-0.397, 0.070)	0.17	-0.578 (-1.051, -0.106)	0.017
Drinking (Reference：Never)						
Never						
Former	0.268 (-0.170, 0.707)	0.229	0.275 (-0.070, 0.621)	0.118	-0.089 (-0.800, 0.623)	0.807
Now	-0.089 (-0.202, 0.024)	0.123	-0.022 (-0.109, 0.064)	0.612	-0.275 (-0.468, -0.083)	0.005
DASH Score	0.001 (-0.012, 0.014)	0.907	0.004 (-0.004, 0.013)	0.315	0.012 (-0.010, 0.034)	0.294
Obesity (Reference：Normal)						
Normal						
Overweight	-0.028 (-0.149, 0.093)	0.646	-0.015 (-0.128, 0.099))	0.799	-0.030 (-0.226, 0.167)	0.767
Obesity	-0.060 (-0.207, 0.087)	0.425	-0.025 (-0.123, 0.073)	0.619	-0.045 (-0.297, 0.206)	0.723
Diabetes (Reference：No)						
No						
Yes	-0.033 (-0.232, 0.166)	0.745	-0.028 (-0.250, 0.195)	0.808	-0.170 (-0.327, -0.012)	0.034
Dyslipidemia (Reference：No)						
No						
Yes	-0.025 (-0.109, 0.058)	0.552	-0.038 (-0.122, 0.046)	0.377	-0.240 (-0.420, -0.061)	0.009

Table S9 The Mediating Role of RTL in the Relationship between OHFS and Hypertension

Variable	Effect	*Estimate* (95% *CI*)	*P*
OHFS (Continuous)			
	Total Effect	0.006 (0.005-0.008)	<0.001
	Direct Effect	0.005 (0.004-0.007)	<0.001
	Indirect Effect	0.001 (0.001-0.001)	<0.001
	Proportion of mediation	16.67%	
OHFS (Categorical)			
Q2 (24.74~)	Total Effect	0.146 (0.040-0.251)	0.007
	Direct Effect	0.121 (0.017-0.224)	0.023
	Indirect Effect	0.025 (0.002-0.048)	0.025
	Proportion of mediation	17.12%	
Q3 (38.98~)	Total Effect	0.181 (0.080-0.283)	<0.001
	Direct Effect	0.139 (0.039-0.240)	0.007
	Indirect Effect	0.042 (0.016-0.064)	<0.001
	Proportion of mediation	23.20%	
Q4 (≥56.58)	Total Effect	0.286 (0.182-0.389)	<0.001
	Direct Effect	0.233 (0.130-0.336)	<0.001
	Indirect Effect	0.052 (0.024-0.072)	<0.001
	Proportion of mediation	18.18%	

The model was adjusted for education level, smoking, drinking, DASH score, obesity, diabetes, and dyslipidemia.

Table S10 The mediating role of RTL in the relationship between occupational hazards with different exposure years and hypertension

Variable	Effect	*Estimate* (95% *CI*)	*P*
Heat			
Low years exposure	Total Effect	0.168 (0.079-0.258)	<0.001
	Direct Effect	0.146 (0.058-0.234)	0.001
	Indirect Effect	0.022 (0.001-0.040)	0.015
	Proportion of mediation	13.10%	
High years exposure	Total Effect	0.222 (0.135-0.309)	<0.001
	Direct Effect	0.182 (0.096-0.268)	<0.001
	Indirect Effect	0.040 (0.020-0.058)	<0.001
	Proportion of mediation	18.01%	
Noise			
Low years exposure	Total Effect	0.128 (0.056-0.199)	0.001
	Direct Effect	0.099 (0.027-0.171)	0.007
	Indirect Effect	0.029 (0.015-0.048)	0.001
	Proportion of mediation	22.66%	
High years exposure	Total Effect	0.158 (0.070-0.245)	<0.001
	Direct Effect	0.108 (0.019-0.197)	0.017
	Indirect Effect	0.050 (0.025-0.065)	<0.001
	Proportion of mediation	31.65%	
Dust			
Low years exposure	Total Effect	0.193 (0.105-0.280)	<0.001
	Direct Effect	0.152 (0.064-0.240)	0.002
	Indirect Effect	0.040 (0.020-0.054)	<0.001
	Proportion of mediation	20.72%	
High years exposure	Total Effect	0.194 (0.103-0.284)	<0.001
	Direct Effect	0.142 (0.052-0.233)	0.002
	Indirect Effect	0.051 (0.027-0.069)	<0.001
	Proportion of mediation	26.29%	
CO			
Low years exposure	Total Effect	0.098 (0.009-0.187)	0.032
	Direct Effect	0.091 (0.004-0.178)	0.040
	Indirect Effect	0.006 (-0.013-0.027)	0.527
	Proportion of mediation	6.12%	
High years exposure	Total Effect	0.180 (0.092-0.268)	<0.001
	Direct Effect	0.147 (0.060-0.234)	0.002
	Indirect Effect	0.033 (0.013-0.054)	0.001
	Proportion of mediation	18.33%	

The model was adjusted for education level, smoking, drinking, DASH score, obesity, diabetes, dyslipidemia, and other occupational hazards apart from the factors under study.

Table S11 The Mediating Role of RTL in the Relationship between OHFS and Hypertension Stratified by Age

Variable	Effect	<45	≥45

		Estimate (95% CI)	P	Estimate (95% CI)	P
OHFS (Continuous)	Total Effect	0.006 (0.005-0.008)	<0.001	0.004 (0.003-0.005)	<0.001
	Direct Effect	0.005 (0.003-0.007)	<0.001	0.003 (0.002-0.004)	<0.001
	Indirect Effect	0.001 (0.000-0.002)	0.008	0.001 (0.001-0.002)	<0.001
	Proportion of mediation	16.67%		25.00%	
OHFS (Categorical)					
Q2 (24.74~)	Total Effect	0.264 (0.124-0.404)	<0.001	0.261 (0.111-0.411)	0.001
	Direct Effect	0.231 (0.093-0.369)	0.001	0.188 (0.040-0.336)	0.013
	Indirect Effect	0.033 (0.002-0.070)	0.001	0.073 (0.027-0.098)	<0.001
	Proportion of mediation	12.50%		27.96%	
Q3 (38.98~)	Total Effect	0.253 (0.107-0.400)	0.001	0.223 (0.075-0.371)	0.003
	Direct Effect	0.192 (0.045-0.340)	0.011	0.175 (0.028-0.322)	0.02
	Indirect Effect	0.061 (0.025-0.092)	<0.001	0.048 (0.012-0.087)	0.009
	Proportion of mediation	24.11%		21.52%	
Q4 (≥56.58)	Total Effect	0.286 (0.093-0.479)	0.004	0.325 (0.186-0.464)	<0.001
	Direct Effect	0.245 (0.055-0.435)	0.012	0.240 (0.102-0.378)	0.001
	Indirect Effect	0.041 (0.004-0.053)	0.001	0.085 (0.040-0.120)	<0.001
	Proportion of mediation	14.34%		26.15%	

The model was adjusted for education level, smoking, drinking, DASH score, obesity, diabetes, and dyslipidemia.

Table S12 Mediation Analysis of the Role of RTL in the Relationship between Occupational Hazard Factors and Hypertension

Variable	Effect	*Estimate* (95% *CI*)	*P*
Heat	Total Effect	0.121 (0.057-0.196)	<0.001
	Direct Effect	0.105 (0.040-0.176)	0.006
	Indirect Effect	0.016 (0.002-0.035)	0.016
	Proportion of mediation	13.22%	
Noise	Total Effect	0.124 (0.057-0.198)	<0.001
	Direct Effect	0.093 (0.026-0.166)	0.010
	Indirect Effect	0.031 (0.014-0.053)	<0.001
	Proportion of mediation	25.00%	
Dust	Total Effect	0.120 (0.054-0.190)	<0.001
	Direct Effect	0.095 (0.030-0.165)	0.004
	Indirect Effect	0.025 (0.009-0.044)	<0.001
	Proportion of mediation	20.83%	
CO	Total Effect	0.113 (0.047-0.188)	<0.001
	Direct Effect	0.099 (0.034-0.169)	0.004
	Indirect Effect	0.015 (0.001-0.031)	0.022
	Proportion of mediation	13.27%	

The model was adjusted for education level, smoking, drinking, DASH score, obesity, diabetes, dyslipidemia, and other occupational hazards apart from the factors under study.

Table S13 Mediation Analysis Stratified by Age

Age		<45 Years		≥45 Years	
		Estimate (95% *CI*)	*P*	*Estimate* (95% *CI*)	*P*
Heat	Total Effect	0.222 (0.118-0.322)	<0.001	0.140 (0.048-0.231)	0.004
	Direct Effect	0.192 (0.090-0.296)	<0.001	0.113 (0.024-0.201)	0.022
	Indirect Effect	0.030 (0.005-0.064)	0.014	0.028 (0.006-0.056)	0.016
	Proportion of mediation	13.51%		20.00%	
Noise	Total Effect	0.187 (0.082-0.285)	<0.001	0.103 (0.005-0.197)	0.038
	Direct Effect	0.157 (0.056-0.254)	0.004	0.064 (0.035-0.097)	0.022
	Indirect Effect	0.030 (0.004-0.063)	0.018	0.039 (0.020-0.083)	<0.001
	Proportion of mediation	16.04%		37.86%	
Dust	Total Effect	0.144 (0.046-0.252)	0.006	0.176 (0.087-0.264)	<0.001
	Direct Effect	0.103 (0.001-0.212)	0.042	0.150 (0.068-0.235)	<0.001
	Indirect Effect	0.041 (0.007-0.085)	0.022	0.025 (0.005-0.053)	0.018
	Proportion of mediation	28.47%		14.20%	
CO	Total Effect	0.139 (0.038-0.250)	0.004	0.111 (0.024-0.210)	0.018
	Direct Effect	0.105 (0.005-0.212)	0.038	0.089 (0.005-0.183)	0.049
	Indirect Effect	0.035 (0.005-0.069)	0.016	0.021 (0.003-0.045)	0.018
	Proportion of mediation	25.18%		18.92%	

The model was adjusted for education level, smoking, drinking, DASH score, obesity, diabetes, dyslipidemia, and other occupational hazards apart from the factors under study.

Table S14 Mediation Analysis of the Role of RTL in the Relationship between OHFS and Hypertension after Excluding Patients with Coronary Heart Disease and Atherosclerosis

Variable	Effect	*Estimate* (95% *CI*)	*P*
OHFS (Continuous)	Total Effect	0.005 (0.004-0.005)	<0.001
	Direct Effect	0.004 (0.003-0.005)	<0.001
	Indirect Effect	0.001 (0.001-0.001)	<0.001
	Proportion of mediation	20.00%	
OHFS (Categorical)			
Q2 (24.74~)	Total Effect	0.154 (0.044-0.264)	0.006
	Direct Effect	0.121 (0.013-0.229)	0.029
	Indirect Effect	0.033 (0.006-0.051)	0.004
	Proportion of mediation	21.43%	
Q3 (38.98~)	Total Effect	0.192 (0.086-0.299)	<0.001
	Direct Effect	0.151 (0.046-0.257)	0.005
	Indirect Effect	0.041 (0.014-0.061)	0.001
	Proportion of mediation	21.35%	
Q4 (≥56.58)	Total Effect	0.295 (0.186-0.403)	<0.001
	Direct Effect	0.241 (0.133-0.349)	<0.001

	Indirect Effect	0.054 (0.022-0.072)	<0.001
	Proportion of mediation	18.31%	

The model was adjusted for education level, smoking, drinking, DASH score, obesity, diabetes, and dyslipidemia.

Table S15 Mediation Analysis After Excluding Patients with Coronary Heart Disease and Atherosclerosis

Variable	Effect	*Estimate* (95% *CI*)	*P*
Heat	Total Effect	0.205 (0.132-0.274)	<0.001
	Direct Effect	0.168 (0.094-0.239)	<0.001
	Indirect Effect	0.037 (0.016-0.062)	<0.001
	Proportion of mediation	18.05%	
Noise	Total Effect	0.122 (0.050-0.192)	<0.001
	Direct Effect	0.078 (0.006-0.149)	0.040
	Indirect Effect	0.043 (0.021-0.068)	<0.001
	Proportion of mediation	35.25%	
Dust	Total Effect	0.153 (0.080-0.223)	<0.001
	Direct Effect	0.113 (0.042-0.185)	<0.001
	Indirect Effect	0.040 (0.019-0.066)	<0.001
	Proportion of mediation	26.14%	
CO	Total Effect	0.091 (0.019-0.163)	0.012
	Direct Effect	0.079 (0.008-0.149)	0.024
	Indirect Effect	0.012 (-0.002-0.031)	0.067
	Proportion of mediation	13.19%	

The model was adjusted for education level, smoking, drinking, DASH score, obesity, diabetes, dyslipidemia, and other occupational hazards except for the factor under study.

Table S16 Mediation Analysis After Excluding New cases of hypertension

Variable	Effect	Estimate (95% CI)	P
OHFS (Continuous)	Total Effect	0.004 (0.003-0.005)	<0.001
	Direct Effect	0.003(0.002-0.004)	<0.001
	Indirect Effect	0.001 (0.001-0.001)	<0.001
	Proportion of mediation	25.00%	
OHFS (Categorical)			
Q2 (24.74~)	Total Effect	0.126 (0.017-0.234)	0.024
	Direct Effect	0.099 (-0.008-0.206)	0.071
	Indirect Effect	0.027 (0.001-0.046)	0.016
	Proportion of mediation	21.43%	
Q3 (38.98~)	Total Effect	0.156 (0.051-0.266)	0.004
	Direct Effect	0.113 (0.008-0.217)	0.034
	Indirect Effect	0.043(0.018-0.064)	0.001
	Proportion of mediation	27.57%	
Q4 (≥56.58)	Total Effect	0.259 (0.152-0.367)	<0.001

Direct Effect	0.208 (0.101-0.315)	<0.001
Indirect Effect	0.051 (0.022-0.070)	<0.001
Proportion of mediation	19.69%	

The model was adjusted for education level, smoking, drinking, DASH score, obesity, diabetes, dyslipidemia, and other occupational hazards except for the factor under study.
